# The repressive role of Arabidopsis H2A.Z in transcriptional regulation depends on AtBMI1 activity

**DOI:** 10.1038/s41467-019-10773-1

**Published:** 2019-06-27

**Authors:** Ángeles Gómez-Zambrano, Wiam Merini, Myriam Calonje

**Affiliations:** Institute of Plant Biochemistry and Photosynthesis (IBVF-CSIC-University of Seville), Avenida Américo Vespucio 49, 41092 Seville, Spain

**Keywords:** Ubiquitylation, Transcriptional regulatory elements, Plant molecular biology, Plant stress responses

## Abstract

H2A.Z variant has emerged as a critical player in regulating plant responses to environment; however, the mechanism by which H2A.Z mediates this regulation remains unclear. In Arabidopsis, H2A.Z has been proposed to have opposite effects on transcription depending on its localization within the gene. These opposite roles have been assigned by correlating gene expression and H2A.Z enrichment analyses but without considering the impact of possible H2A.Z post-translational modifications. Here, we show that H2A.Z can be monoubiquitinated by the PRC1 components AtBMI1A/B/C. The incorporation of this modification is required for H2A.Z-mediated transcriptional repression through a mechanism that does not require PRC2 activity. Our data suggest that the dual role of H2A.Z in regulating gene expression depends on the modification that it carries, while the levels of H2A.Z within genes depend on the transcriptional activity.

## Abstract (web summary)

Arabidopsis H2A.Z plays an important role in regulating gene expression in response to stressors; however, the underlying mechanism is still puzzling. Here, the authors show that monoubiquitination of H2A.Z by AtBMI1 is required for H2A.Z-mediated transcriptional repression.

## Introduction

Eukaryotic organisms must respond to environmental changes in order to survive. This response involves changes in gene expression in which chromatin structure plays a central role. Chromatin organization can be altered through the incorporation of histone variants that, unlike canonical histones, occurs independently of replication. This different mechanism of deposition makes them adaptable to respond to environmental stimuli^1^. According to this, H2A.Z variant has emerged as a critical player in regulating plant responses to environment. Several reports have demonstrated that Arabidopsis H2A.Z plays a role in regulating gene expression in response to temperature fluctuations, drought, pathogen infection, and nutrient stress^1–7^. Nevertheless, in addition to its role in transcriptional regulation, H2A.Z in plants has been related to other processes, such as DNA repair, somatic homologous recombination, and replication origin specification^8^.

H2A.Z in Arabidopsis, as in other organisms, frequently localizes immediately downstream transcription start sites, co-localizing with +1 nucleosome^9–12^, but it is also found further within gene bodies^13^. H2A.Z has been proposed to have either a promoting or repressive effect on transcription depending on its localization within the gene. The presence of H2A.Z in +1 nucleosome has been associated with transcriptional activation, while its presence in nucleosomes further within gene bodies is associated with transcriptional repression and positively correlated with gene responsiveness^7,13^. In contrast, a recent report showed that H2A.Z enrichment at +1 nucleosome is required to repress gene expression^14^; therefore, the role of H2A.Z is still puzzling.

A reason for these conflicting results could be that the incorporation of H2A.Z has been examined as a whole without considering posttranscriptional modifications, which provides an incomplete picture to understand the role of this histone variant. Here, we show that H2A.Z can be monoubiquitinated by the PRC1 components AtBMI1A/B/C^15,16^. The incorporation of this modification is required for H2A.Z-mediated transcriptional repression. Moreover, our data indicate that more than H2A.Z incorporation H2A.Z monoubiquitination is what is decisive for H2A.Z-mediated repression.

## Results

### A modified H2A.Z is required to regulate flowering time

In Arabidopsis, there are three functional genes encoding H2A.Z, *HTA8*, *HTA9*, and *HTA11*^17^. Expression analysis of the three genes in wild-type Col-0 (WT) seedlings at 7 days after germination (DAG) showed that *HTA9* displayed the highest expression levels while *HTA8* the lowest (Supplementary Fig. 1a). Both double and triple mutants are viable, although the latter shows severer developmental defects^13^. The most obvious trait in *hta9hta11* mutants is their early-flowering phenotype^2^, a similar phenotype to the one displayed by mutant plants in components of the SWI2/SNF2-related complex (SWR1C), which is involved in the incorporation of H2A.Z^8^. Accordingly, the levels of the flowering-promoter *FLOWERING LOCUS T* (*FT*) are increased in *hta9hta11* and those of the flowering-repressor *FLOWERING LOCUS C* (*FLC)* are reduced^2^.

**Fig. 1:**
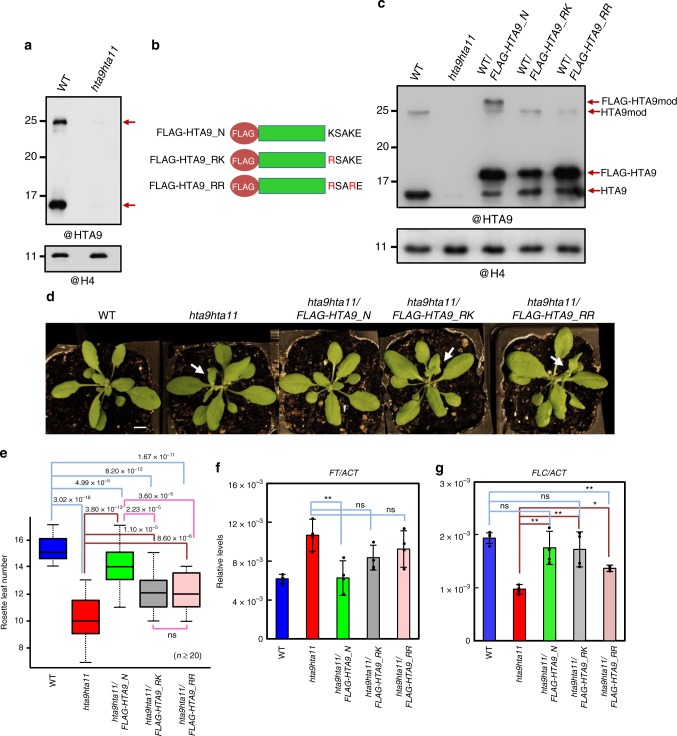
A modified form of H2A.Z is required to regulate flowering time. **a** Western blot (WB) analysis of HTA9 levels in histone enriched extracts of wild-type (WT) and *hta9hta11* mutants at 7 days after germination (DAG; upper panel). The same blot was probed with anti-H4 antibody for loading control (bottom panel). Arrows indicate the bands recognized by the antibody. **b** Schematic representation of the C-terminal region of FLAG-tagged *HTA9* native construct (*FLAG-HTA9_N*) or mutated in which K129 or both K129 and K132 were replaced by R (*FLAG-HTA9_RK* and *FLAG-HTA9_RR*, respectively). **c** WB analysis of HTA9 and FLAG-HTA9 levels in WT, *hta9hta11*, *WT*/*FLAG-HTA9_N*, *WT*/*FLAG-HTA9_RK*, and *WT*/*FLAG-HTA9_RR* (upper panel). Arrows indicate the bands recognized by the antibody. The same blot was probed with anti-H4 antibody for loading control (bottom panel). **d** Picture showing WT, *hta9hta11*, *hta9hta11/FLAG-HTA9_N*, *hta9hta11/FLAG-HTA9_RK*, and *hta9hta11/FLAG-HTA9_RR* plants at 24 DAG growing under long day conditions. Arrows indicate the presence of elongated shoot. Bar indicates 1 cm. **e** Box plots indicating the number of rosette leaves at bolting in different genotypes. “*n*” indicates the number of plants. In each case, the median (segment inside rectangle), upper and lower quartiles (boxes), and minimum and maximum values (whiskers) are indicated. *p* Value of significant differences as determined by Student’s *t* test are indicated. **f** Relative *FT* expression levels in the different genotypes at 12 DAG. *ACTIN2* (*ACT2*) was used as an internal control. Error bars indicate standard deviation of *n* = 4 biological replicates. Data points are indicated in the bar charts. **g** Relative *FLC* expression levels in different genotypes at 12 DAG. *ACT*2 was used as an internal control. Error bars indicate standard deviation of *n* = 4 biological replicates. Data points are indicated in the bar charts. Significant differences as determined by Student’s *t* test are indicated (**P < 0.01; *P < 0.05; ns, not significant). Source data of Fig. 1a, c, e–g are provided as a Source Data file

Western blot (WB) analysis of WT and *hta9hta11* histone extracts at 7 DAG using anti-HTA9 antibody not only confirmed reduced levels for HTA9-predicted band in *hta9hta11* (Fig. 1a) but also revealed the presence of a band of around 25 kDa whose molecular weight (MW) might be consistent with the addition of one ubiquitin. This band represented 20–30% of total HTA9 in WT (Supplementary Fig. 1b), indicating that a minor fraction of H2A.Z might carry this modification. To investigate whether the band was indeed a modified HTA9, we generated WT transgenic lines expressing an N-terminal FLAG-tagged version of *HTA9* (*FLAG-HTA9_N*) under the control of *CaMV35s* promoter (Fig. 1b). Anti-HTA9 antibody recognized the unmodified and modified forms of both HTA9 and FLAG-HTA9_N on histone extracts from these plants (Fig. 1c), confirming that the 25 kDa band was a modified HTA9.

H2A.Z monoubiquitination (H2A.Zub) marks have been detected in mammalian embryonic stem cells at bivalent genes but they decrease upon differentiation^18,19^. This modification occurs mostly at lysine (K) 120 and to a lesser extent at K121 and K125^18^. Thus we compared the protein sequences of HTA9 and human H2A.Z and selected K129 and K132 as possible target lysine/s for monoubiquitination in Arabidopsis (Supplementary Fig. 1c). We then generated WT transgenic plants constitutively expressing a *FLAG-HTA9* version in which K129 or both K129 and K132 were replaced by arginine (R) (WT/*FLAG-HTA9_RK* and WT/*FLAG-HTA9_RR*, respectively) (Fig. 1b). These mutations resulted in a complete loss of the modified FLAG-HTA9 band as determined by WB on histone extracts (Fig. 1c), which suggests that K129 is the predominant target lysine for this modification, although it cannot be ruled out that K132 is also modified to some extent. To explore whether the different constructs were able to rescue *hta9hta11* mutant defects, we introduced *FLAG-HTA9_N*, *FLAG-HTA9_RK*, or *FLAG-HTA9_RR* into *hta9hta11* plants, and after verifying that the proteins were expressed (Supplementary Fig. 2b) and incorporated into chromatin by WB analysis after chromatin immunoprecipitation (ChIP) using anti-H3 antibody (Supplementary Fig. 2c), we examined the phenotype of different plants. We found that the flowering time of *hta9hta11/FLAG-HTA9_N* plants was delayed compared to *hta9hta11*, *hta9hta11/FLAG-HTA9_RK* and *hta9hta11/FLAG-HTA9_RR* (Fig. 1d, e), which was supported by *FT* transcript levels (Fig. 1f); however, we noticed that the early flowering phenotype of *hta9hta11* was partially rescued in the three lines, although *hta9hta11/FLAG-HTA9_N* showed a greater rescue (Fig. 1e). Since the early flowering phenotype of *hta9hta11* is in part a consequence of reduced *FLC* expression levels^2^, we checked the levels of *FLC* in those plants (Fig. 1g). We found that *FLC* levels were increased in the three lines compared to *hta9hta11*. Interestingly, despite the similar levels of *FLC* expression in *hta9hta11/FLAG-HTA9_N* and *hta9hta11/FLAG-HTA9_RK*, *FT* expression in *hta9hta11/FLAG-HTA9_RK* was not as low as in *hta9hta11/FLAG-HTA9_N*, indicating that both the incorporation of H2A.Z and the establishment of the modification are required to control flowering time.

### The AtBMI1 proteins mediate H2A.Z monoubiquitination

**Fig. 2:**
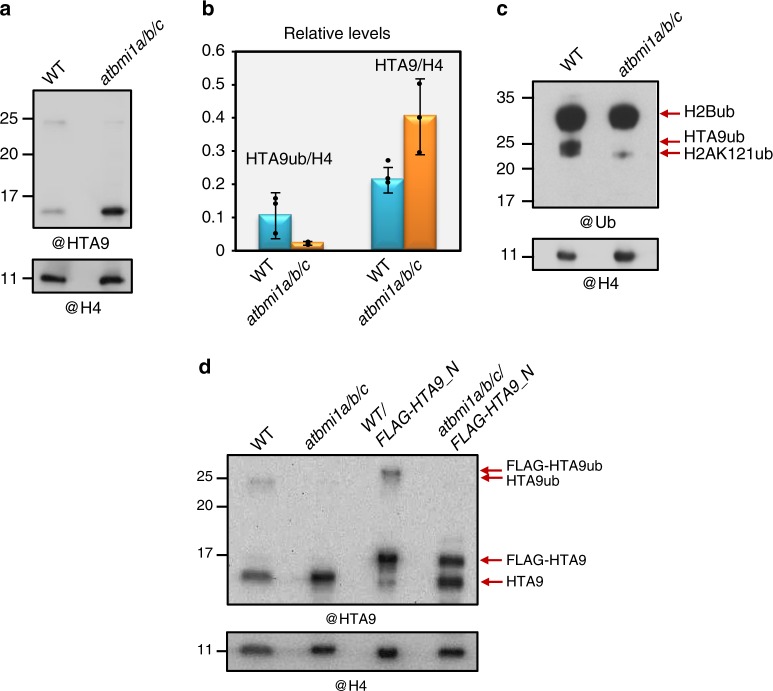
The AtBMI1 proteins mediate H2A.Z monoubiquitination. **a** Western blot (WB) analysis of HTA9 levels in histone-enriched extracts of wild-type (WT) and *atbmi1a/b/c* mutant at 7 days after germination (DAG). The same blot was probed with anti-H4 antibody for loading control. **b** Quantification of HTA9 and HTA9ub levels in WT and *atbmi1a/b/c* mutant relative to H4; Error bars indicate standard deviation of *n* = 3 independent experiments. Data points are indicated in the bar charts. **c** WB analysis of WT and *atbmi1a/b/c* mutant histone-enriched extracts at 7 DAG probed with anti-ubiquitin antibody. Bands corresponding to H2Bub, H2AK121ub, and HTA9ub are indicated. A blot probed with anti-H4 antibody was used as a loading control. **d** WB analysis of HTA9 and FLAG-HTA9 levels in WT, *atbmi1a/b/c*, *WT/FLAG-HTA9_N*, and *atbmi1a/b/c/FLAG-HTA9_N*. Arrows indicate the bands recognized by the antibody. The same blot was probed with anti-H4 antibody for loading control. Source data of Fig. 2a–d are provided as a Source Data file

Vertebrate Polycomb Repressive Complex 1 (PRC1) mediates H2A and H2A.Z monoubiquitination^18,20^; however, the PRC1 monoubiquitin ligase module in Arabidopsis, which comprises one AtBMI1 (AtBMI1A/B/C) and one AtRING1 (AtRING1A/B) protein^15,21,22^, has so far only been involved in canonical H2A lysine 121 monoubiquitination (H2AK121ub)^21^. This activity is required to maintain gene repression throughout plant development^23,24^. *atbmi1a/b/c* mutant remains in an embryo maturating-like phase due to misexpression of the embryonic program after germination^25^ (Supplementary Fig. 3). Moreover, 20% of the Arabidopsis transcriptome is misregulated in this mutant^24^. PRC1 acts together with PRC2, which mediates H3 lysine 27 trimethylation (H3K27me3)^26–30^, in the repression of around 5000 genes^31^. In fact, PRC2 requires the presence of H2AK121ub marks to incorporate H3K27me3 at these genes^31^. Accordingly, *atbmi1a/b/c* mutant displays strongly reduced levels of H2AK121ub and H3K27me3^31^. Therefore, to investigate whether AtBMI1 activity is involved in H2A.Z monoubiquitination, we compared the levels of modified HTA9 in WT and *atbmi1a/b/c* (Fig. 2a, b) and we found that the signal was at least five times reduced in mutant seedlings. In addition, we found that anti-ubiquitin antibody could recognize modified HTA9 in WT but not in *atbmi1a/b/c* (Fig. 2c) and that the antibody recognized a band with the MW of HTA9ub after immunoprecipitation of WT chromatin with anti-HTA9 antibody (Supplementary Fig. 3a). Furthermore, when the *FLAG-HTA9_N* construct was introduced into *atbmi1a/b/c* mutant, the FLAG-HTA9ub band could not be detected (Fig. 2d). All together, these results indicate that AtBMI1 activity is required for H2A.Z monoubiquitination.

### H2A.Zub has a repressive role in transcription

**Fig. 3:**
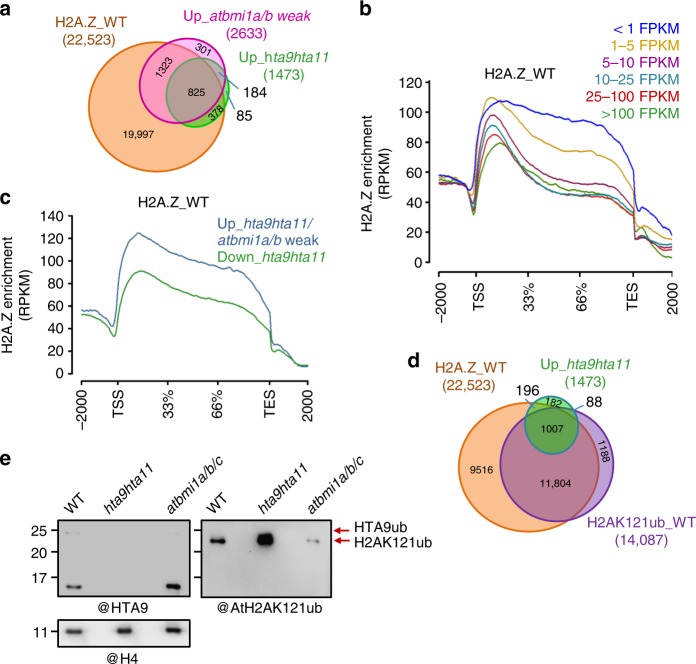
Overlap between upregulated genes in *hta9hta11* and *atbmi1a/b*. **a** Venn diagram showing the number of overlapping genes when comparing H2A.Z-enriched genes in wild-type (WT) seedlings (H2A.Z_WT), upregulated genes in *hta9hta11*, and upregulated genes in *atbmi1a/b* weak mutant. **b** Metagene plots showing H2A.Z levels over different subsets of genes grouped according to their expression levels (indicated in FPKM (fragments per kilobase of exon and million mapped reads) and different colors). TSS transcription start site, TES transcription stop site. **c** Metagene plots showing H2A.Z enrichment in WT at genes upregulated in *hta9hta11* and *atbmi1a/b* weak mutants and at genes downregulated in *hta9hta11*. **d** Venn diagram showing the number of overlapping genes when comparing H2A.Z-enriched genes in WT (H2A.Z_WT), H2AK121ub marked genes in WT (H2AK121ub_WT), and upregulated genes in *hta9hta11*. **e** WB blot showing H2AK121ub and HTA9ub levels in WT, *hta9hta11*, and *atbmi1a/b/c* (right and left panels, respectively). The blot was probed with an anti-AtH2AK121ub antibody, then stripped and re-probed with anti-HTA9 antibody. Arrows indicate the bands recognized by the antibody. The same blot was probed with anti-H4 antibody for loading control (bottom panel). Source data of Fig. 3e are provided as a Source Data file

To investigate the role of H2A.Zub in regulating gene expression, we first compared the genes enriched in H2A.Z in WT^32^ (H2A.Z_WT; Supplementary Data 1) with the genes misregulated in *hta9hta11* and *atbmi1a/b* weak mutants compared to WT at 7 DAG (log2-fold change cut-off >|1|, *p* value <0.05 according to a moderated *t* test; Supplementary Data 2). We used *atbmi1a/b* weak mutant as it can switch to vegetative development after germination^21^ and thus displays a phenotype comparable to *hta9hta11* (Supplementary Fig. 3). We found that most of the upregulated genes in *hta9hta11* and *atbmi1a/b* weak mutants overlapped with the group of genes enriched in H2A.Z in WT. Interestingly, the majority of the genes upregulated in *hta9hta11* were also upregulated in *atbmi1a/b* weak mutant (Fig. 3a), indicating that perhaps H2A.Zub mediates this repression.

Analysis of H2A.Z enrichment at genes grouped according to their expression levels in WT seedlings (Fig. 3b) showed that both active and repressed genes were enriched in H2A.Z, which is in agreement with previous reports^7,13^. H2A.Z enrichment was not only observed at +1 nucleosome region but also along gene bodies, although H2A.Z levels were negatively correlated with expression levels (Fig. 3b). We found that, in WT seedlings, the genes upregulated in *hta9hta11* and *atbmi1a/b* weak mutants showed higher H2A.Z levels along the entire gene than the genes downregulated in *hta9hta11* (Fig. 3c), which was consistent with a transcriptionally repressed and activated state, respectively.

According to our results, AtBMI1 proteins are involved in both H2A and H2A.Z monoubiquitination. Since the incorporation of H2AK121ub marks have a repressive role, we compared the genes enriched in H2A.Z in WT with the ones marked with H2AK121ub also in WT^31^ and the ones upregulated in *hta9hta11* (Supplementary Data 1; Supplementary Data 2; Fig. 3d). We found that 1007 out of the 1473 upregulated genes in *hta9hta11* were enriched in H2A.Z and marked with H2AK121ub in WT, indicating that H2A.Z and H2AK121ub marks co-occupy most of the genes that became upregulated in *hta9hta11*. Therefore, we investigated whether the levels of H2AK121ub were altered in *hta9hta11* mutants using an Arabidopsis anti-H2AK121ub-specific antibody^25^. Interestingly, H2AK121ub levels were increased in *hta9hta11* mutants (Fig. 3e). Thus it might be possible that in the absence of H2A.Z AtBMI1 activity tries to compensate for the loss of H2A.Zub by increasing monoubiquitination of canonical H2A; however, the fact that *hta9hta11* mutant displayed misregulation of a considerable number of genes (1473 upregulated and 1702 downregulated; Supplementary Data 2) indicates that H2AK121ub cannot replace the role of H2A.Zub in regulating these genes, which indicates independent roles.

**Fig. 4:**
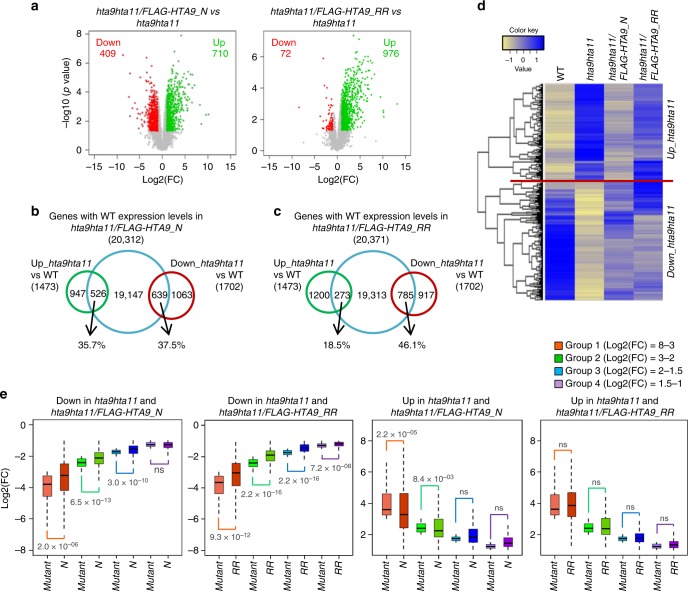
H2A.Z monoubiquitination has a repressive role in gene regulation. **a** Volcano plots showing downregulated (left, red dots) and upregulated (right, green dots) genes in *hta9hta11/FLAG-HTA9_N* (left panel) or *hta9hta11/FLAG-HTA9_RR* (right panel) compared to *hta9hta11*. **b**, **c** Comparison of the genes upregulated and downregulated in *hta9hta11* according to wild-type (WT) levels and the genes with WT expression levels in *hta9hta11/FLAG-HTA9_N* (**b**) or *hta9hta11/FLAG-HTA9_RR* (**c**). The percentages of upregulated and downregulated genes in *hta9hta11* that recovered WT expression levels in each line are indicated. **d** Heatmap representing the expression levels of the genes misregulated in *hta9hta11* in different genotypes. Color code represents normalized expression values measured in FPKM (fragments per kilobase of exon and million mapped reads) (pale yellow is the lowest and dark blue the highest expression levels). A standard normalization of the gene expression profiles was performed to obtain a mean expression of 0 and a standard deviation of 1 in order to make the expression profiles comparable. **e** Box plots showing differential expression (DE, log2-fold change) relative to WT of the genes commonly upregulated and downregulated in *hta9hta11* and *hta9hta11/FLAG-HTA9_N* or in *hta9hta11* and *hta9hta11/FLAG-HTA9_RR*. Upregulated or downregulated genes in *hta9hta11* were divided into four groups (200–300 genes in each group) according to their DE and compared to the DE that they displayed in *hta9hta11/FLAG-HTA9_N* or *hta9hta11/FLAG-HTA9_RR*. In each case, the median (segment inside rectangle), upper and lower quartiles (boxes), and minimum and maximum value (whiskers) were indicated. *p* Value according to Wilcoxon test of significant differences between mutant and *hta9hta11/FLAG-HTA9_N* (*N*) or *hta9hta11/FLAG-HTA9_RR* (*RR*) were indicated; ns indicates no significant difference

We next compared the transcriptome of *hta9hta11/FLAG-HTA9_N* or *hta9hta11/FLAG-HTA9_RR* to that of *hta9hta11* mutant at 7 DAG (Supplementary Data 3) to investigate the genes that change their expression levels in *hta9hta11* as a consequence of *FLAG-HTA9_N* or *FLAG-HTA9_RR* expression. Volcano plots representing differentially expressed genes (DEGs; log2-fold change cut-off >|1|, *p* value <0.05; Fig. 4a) revealed that, in both *hta9hta11/FLAG-HTA9_N* and *hta9hta11/FLAG-HTA9_RR*, a considerable number of genes became upregulated compared to *hta9hta11* (710 and 976, respectively); however, the number of downregulated genes in *hta9hta11/FLAG-HTA9_N* was higher than in *hta9hta11/FLAG-HTA9_RR* (409 and 72, respectively). Consistent with this, we found that a high percentage of the genes downregulated in *hta9hta11* recovered their WT expression levels in both *hta9hta11/FLAG-HTA9_N* and *hta9hta11/FLAG-HTA9_RR* plants (37.5% and 46.1%, respectively; Fig. 4b, c; Supplementary Fig. 4a, b); however, the percentage of upregulated genes in *hta9hta11* that recovered their WT levels was considerably lower in *hta9hta11/FLAG-HTA9_RR* than in *hta9hta11/FLAG-HTA9_N* (18.5% and 35.7%, respectively; Fig. 4b, c; Supplementary Fig. 4a, b).

Nevertheless, using our cut-off criteria, there were still a high number of genes commonly upregulated and downregulated in *hta9hta11* and *hta9hta11/FLAG-HTA9_N* or *hta9hta11/FLAG-HTA9_RR* (943 or 1200 upregulated, respectively, and 1041 or 880 downregulated, respectively; Supplementary Fig. 4c). However, when we compared the expression levels of the genes upregulated and downregulated in *hta9hta11* in different genotypes (Fig. 4d), we found that, in general, the expression levels of the upregulated genes in *hta9hta11/FLAG-HTA9_N* were more similar to WT than in *hta9hta11/FLAG-HTA9_RR*, while the expression levels of downregulated genes were partially recovered in the two lines. Therefore, to further investigate a possible change in the expression of the genes commonly misregulated in *hta9hta11* and *_N* or *_RR* lines (Supplementary Fig. 4c), we compared the differential expression (DE) relative to WT displayed by these genes in different genotypes (Supplementary Data 4). For this, we divided the commonly upregulated or downregulated genes in each comparison in four groups (200–300 genes in each group) according to their DE in *hta9hta11* and investigated their DE in *hta9hta11/FLAG-HTA9_N* or *hta9hta11/FLAG-HTA9_RR* (Fig. 4e). We found that the DE of downregulated genes was significantly reduced in both *hta9hta11/FLAG-HTA9_N* and *hta9hta11/FLAG-HTA9_RR* compared to *hta9hta11*, showing a tendency to recover WT expression levels, which fitted with the expression levels of *FLC* found in these lines (Fig. 1g). However, the DE of upregulated genes was only significantly reduced in *hta9hta11/FLAG-HTA9_N*, as was the case of *FT* expression (Fig. 1f). The expression of *FT* has been recently shown to be directly modulated by H2A.Z deposition^33^. All together, these data not only argue for a repressive role of HTA9ub but also indicate that incorporation of either native or mutated HTA9 can mediate gene activation, which supports the dual role of H2A.Z in gene regulation^7,13^.

### H2A.Z-mediated repression is independent of PRC2

**Fig. 5:**
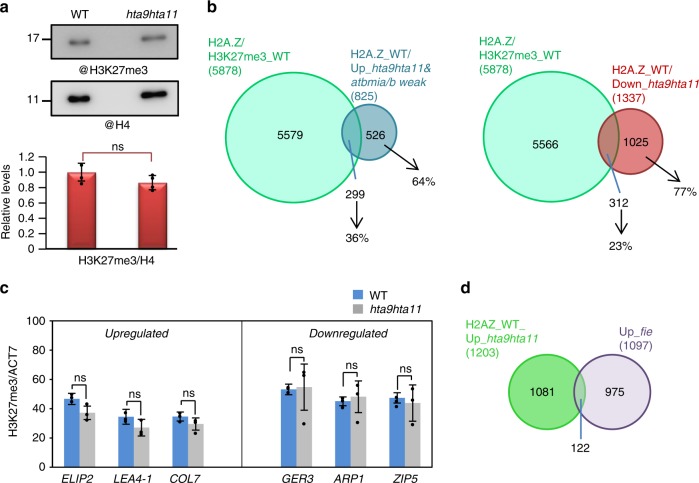
H2A.Zub repressive effect is independent of Polycomb Repressive Complex 2 activity. **a** Western blot showing global H3K27me3 levels in wild-type (WT) and *hta9hta11* mutants at 7 days after germination (DAG). The same blot was probed with anti-H4 antibody for loading control. Bottom bar chart shows quantification of H3K27me3 levels relative to H4. Error bars indicate standard deviation of *n* = 4 independent experiments. Data points are indicated in the bar charts. No significant (ns) differences between WT and *hta9hta11* were found according to Student’s *t* test. **b** Left panel, Venn diagram showing the percentage of upregulated genes in *hta9hta11* and *atbmi1a/b* weak that were enriched in H2A.Z and H3K27me3 marked in WT (H2A.Z/H3K27me3_WT) at 7 DAG. Right panel, Venn diagram showing the overlap between genes downregulated in *hta9hta11* and H2A.Z/H3K27me3_WT genes. **c** H3K27me3 levels analyzed by chromatin immunoprecipitation–quantitative polymerase chain reaction at ATG surrounding region of several upregulated and downregulated genes in *hta9hta11* that were H2A.Z/H3K27me3_WT (genes from overlaps of **b**). The levels were normalized to *ACTIN7* (*ACT7*). Error bars indicate standard deviation of *n* = 3 biological replicates. Data points are indicated in the bar charts. No significant (ns) differences between WT and *hta9hta11* were found according to Student’s *t* test. **d** Venn diagram showing the overlap between the H2A.Z-enriched genes that are upregulated in *hta9hta11* and the ones upregulated in *fie* mutants. Source data of Fig. 5a, c are provided as a Source Data file

Recent reports in plants and animals have showed that canonical H2A monoubiquitination is needed for H3K27me3 marking at a considerable number of genes^25,31,34–36^; however, there are also genes only marked with H2AK121ub^31,37^ or H3K27me3^31^. Also, H3K27me3 and H2A.Z in vertebrates have been shown to co-localize at bivalent genes in embryonic stem cells and that knockdown *h2a.z* mutants displayed decreased levels of H3K27me3^18,38^. Moreover, H2A.Zub has been proposed to modulate H3K27me3 deposition at bivalent genes^19^, whereas H2A.Z is absent from H3K27me3-enriched genes that are stably repressed^18^. Co-localization of H2A.Z and H3K27me3 has been also reported in plants at a subset of genes^39,40^ and a role of H2A.Z in modulating H3K27me3 marking has been proposed^14,39^. Therefore, we wondered whether H2A.Zub could be required for H3K27me3 marking. According to previous data^39^, we found that almost all H3K27me3 marked genes (H3K27me3_WT) in WT seedlings^31^ (Supplementary Data 1) were enriched in H2A.Z (Supplementary Fig. 5a), which was not surprising since H2A.Z was detected at 22,523 genes in WT seedling^32^ (Supplementary Data 1); however, when we analyzed the global levels of H3K27me3 in *hta9hta11* mutants, we did not find significant changes in mutant compared to WT (Fig. 5a). A similar result was obtained when analyzing the global levels of H3K27me3 in the SWR1C mutant *actin-related protein 6–10* (*arp6–10*)^4^ (Supplementary Fig. 5a). We then determined the percentage of genes enriched in H2A.Z and marked with H3K27me3 in WT (H2A.Z/H3K27me3_WT) that were upregulated in *hta9hta11* and *atbmi1a/b* weak mutants and enriched in H2A.Z in WT (to consider only H2A.Z direct target genes, Fig. 5b, left panel). In all, 36% of the upregulated genes (299 out of 825) were H3K27me3 marked, while the remaining 64% was not. We also found little overlap between the H2A.Z/H3K27me3_WT genes and the genes downregulated in *hta9hta11* (Fig. 5b, right panel), indicating that most of the genes enriched in H2A.Z whose expression is altered in *hta9hta11* mutants are not PRC2 targets. To investigate whether the H2A.Z/H3K27me3_WT marked genes displayed altered levels of H3K27me3 in *hta9hta11* mutants, we selected three upregulated and three downregulated genes among the overlapping genes (Fig. 5b; Supplementary Fig. 5b) and analyzed their H3K27me3 levels by ChIP–quantitative polymerase chain reaction (qPCR); however, we did not find significant changes at any of these genes (Fig. 5c). Nevertheless, this result cannot rule out a possible role of H2A.Zub in modulating H3K27me3 deposition at other loci.

We next wondered whether the combined action of H2A.Zub and H3K27me3 is required to regulate the expression of H2A.Z/H3K27me3_WT genes. Therefore, we compared the genes upregulated in the PRC2 mutant *fertilization independent endosperm* (*fie*), in which H3K27me3 deposition is abolished genome wide^28^, and in *hta9hta11*, but we found that only a 10% of the genes in each set overlapped in this comparison (Fig. 5d). Therefore, considering that most of the upregulated genes in *hta9hta11* and enriched in H2A.Z in WT were not marked with H3K27me3 in WT (64%), and the little overlap found between the upregulated genes in *hta9hta11* and *fie* mutants, it seems that, despite H2A.Z may co-localize with H3K27me3 at a subset of targets and may modulate the H3K27me3 levels, the transcriptional-repressive effect of H2A.Zub is not dependent on PRC2 activity.

### H2A.Z levels at genes depends on transcriptional activity

**Fig. 6:**
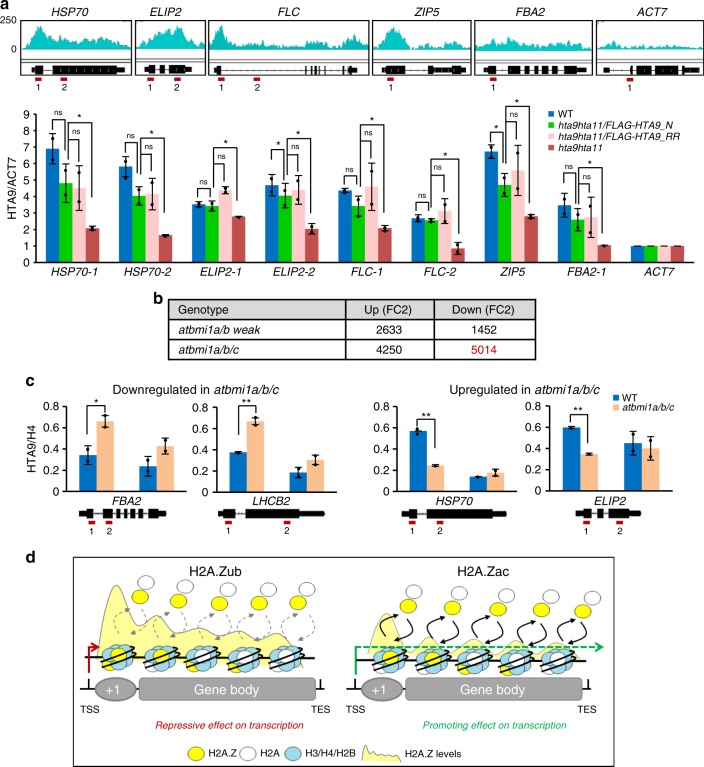
H2A.Zub more than H2A.Z incorporation is required for repression. **a** HTA9 levels analyzed by chromatin immunoprecipitation–quantitative polymerase chain reaction (ChIP-qPCR) at different genes in wild-type (WT), *hta9hta11/FLAG-HTA9_N*, *hta9hta11/FLAG-HTA9_RR* and *hta9hta11* plants at 7 days after germination (DAG). Browser views of H2A.Z levels in WT at the selected genes and schematic representation of each gene with red lines indicating the position of amplified regions are shown. HTA9 levels were normalized to *ACT7*. Error bars indicate standard deviation of *n* = 2 biological replicates. Data points are indicated in the bar charts. Significant differences as determined by Student’s *t* test are indicated (*P < 0.05; ns, not significant). **b** Number of genes upregulated and downregulated determined by RNA-seq in *atbmi1a/b* weak and *atbmi1a/b/c* compared to WT seedlings at 7 DAG. Downregulated genes in *atbmi1a/b/c* are highlighted in red. **c** HTA9 levels analyzed by ChIP-qPCR at two downregulated and two upregulated genes in *atbmi1a/b/c* (Supplementary Fig. 6b, c). A schematic representation of the genes is included indicating the position of amplified regions (red lines). HTA9 levels were normalized to H4 levels. Error bars indicate standard deviation of *n* = 2 biological replicates. Data points are indicated in the bar charts. Significant differences as determined by Student’s *t* test are indicated (*P < 0.05; **P < 0.01). **d** Proposed model for H2A.Z-mediated gene regulation in which monoubiquitination of H2A.Z is required for transcriptional repression (left) and acetylation of H2A.Z for transcriptional activation, while H2A.Z levels along the genes are consequence of the transcriptional activity, where transcriptional repression leads to accumulation of H2A.Z (left) and active transcription to decreased levels of H2A.Z (right). Source data of Fig. 6a, c are provided as a Source Data file

The incorporation of H2A.Z along genes has been proposed to have a repressive role in transcription^7,13^. In line with this, we found a negative correlation between gene expression levels and H2A.Z enrichment along genes (Fig. 3b); Moreover, the genes that became upregulated in *hta9hta11* and *atbmi1a/b* weak were enriched in H2A.Z in WT seedlings compared to those that become downregulated in *hta9hta11* (Fig. 3c). However, according to expression analyses in *hta9hta11*, *hta9hta11/FLAG-HTA9_N*, and *hta9hta11/FLAG-HTA9_RR* plants, the number of upregulated genes in *hta9hta11* that recovered their WT-like expression levels was much higher when FLAG-HTA9_N was introduced than when FLAG-HTA9_RR was. Therefore, we wondered whether the incorporation of H2A.Z was indeed an active mechanism to repress transcription or a consequence of gene activity. To evaluate this, we checked whether the two HTA9 variants (FLAG-HTA9_N and FLAG-HTA9_RR) were similarly incorporated into genes whose expression is recovered or partially recovered in one or in the two lines (Fig. 6a). We selected two well-known H2A.Z targets, *FLC* and *HEAT SHOCK PROTEIN 70* (*HSP70*), in which the incorporation of H2A.Z has been shown to have a promoting and a repressive effect in transcription, respectively^2,4,6,8,41^. Consistent with this, WT-like expression levels of *FLC* were recovered in both lines (Fig. 1g), while *HSP70* WT levels were recovered only in *hta9hta11/FLAG-HTA9_N* (Supplementary Fig. 4a). We included in the analysis other genes whose expression was upregulated (*EARLY LIGHT-INDUCED PROTEIN 2* (*ELIP2*)) or downregulated (*ZINC TRANSPORTER 5* (*ZIP5*); *FRUCTOSE-BISPHOSPHATE ALDOLASE 2* (*FBA2*)) to different extents in *hta9hta11* and also *ACTIN 7* (*ACT7*) as a negative control for H2A.Z incorporation (Fig. 6a). We found that FLAG-HTA9_N and FLAG-HTA9_RR were similarly incorporated at the chromatin of these genes and that, despite the levels of HTA9 did not reach WT levels at some of the investigated regions, the levels of HTA9 in both lines were significantly higher than in *hta9hta11*, suggesting that H2A.Z monoubiquitination, more than H2A.Z incorporation, is what is decisive for H2A.Z-mediated repression.

Interestingly, we noticed that HTA9 levels were significantly increased in *atbmi1a/b/c* compared to WT (Figs. 2a, d and 3e), even considering the monoubiquitinated fraction included. *atbmi1a/b* weak and *atbmi1a/b/c* mutants displayed a very high number of upregulated genes (Fig. 6b; Supplementary Data 2, and Supplementary Data 5), which is consistent with a loss of PRC1 repression. However, unlike *atbmi1a/b* weak mutant, *atbmi1a/b/c* mutant showed a higher number of downregulated genes than upregulated (Fig. 6b; Supplementary Data 2, and Supplementary Data 5), which might be a consequence of the lack of tissue-specific activating factors required for the expression of these genes in this embryo-like mutant, as most of these genes are properly expressed in *atbmi1a/b* weak mutant (Supplementary Fig. 6a). We compared the levels of HTA9 in *atbmi1a/b/c* and WT at two genes that became repressed in *atbmi1a/b/c* but not in *atbmi1a/b* weak mutant and at two genes that became upregulated in both *atbmi1* mutants (Supplementary Fig. 6b, c). We found that the levels of HTA9 were significantly increased at the repressed genes in *atbmi1a/b/c* (Fig. 6c, left) while decreased at genes that became upregulated (Fig. 6c, right). Therefore, the high number of downregulated genes in *atbmi1a/b/c* might explain the globally increased levels of HTA9 in mutant compared to WT. Furthermore, this result supports the hypothesis that the accumulation of H2A.Z at genes depends on their transcriptional activity, as HTA9 is incorporated to a higher extent at those genes that could not be properly activated in *atbmi1a/b/c*.

## Discussion

H2A.Z enrichment at the +1 nucleosome has been proposed to be required for both transcriptional repression and activation, while its presence in nucleosomes further within gene bodies has been related with transcriptional repression^7,13,14^. However, we found that H2A.Z monoubiquitination plays an important role in the transcriptional repression mediated by this histone variant, emerging as a key factor to understand H2A.Z function in gene regulation.

Complementation of *hta9hta11* mutants with a HTA9 form susceptible for monoubiquitination was able to recover or partially recover WT expression levels of most of the genes upregulated in *hta9hta11*, while this effect was much lower when *hta9hta11* was complemented with a HTA9 form unable to be monoubiquitinated. The partially rescued expression levels of upregulated genes in *hta9hta11/FLAG-HTA9_N* might be a consequence of the FLAG-tag fusion at the N-terminal, which might be interfering to some extent with the proper incorporation of this variant into chromatin. We showed that H2A.Z monoubiquitination is mediated by PRC1. Loss-of-function of AtBMI1 proteins led to reduced levels of both HTA9 and H2A monoubiquitination; accordingly, the upregulated genes in *hta9hta11* mutants represented a subset of the ones upregulated in *atbmi1* mutants. Although we could not determine the localization pattern of H2A.Zub at target genes due to the lack of specific antibodies, the incorporation of one ubiquitin at the C-terminal region of H2A.Z, either at +1 nucleosome region or at any location within gene body, might change its biochemical properties leading to an increase in nucleosome stability that impedes transcription. Alternatively, H2A.Zub might interact with specific chromatin-associated proteins that affect gene expression as has been proposed before^19^.

Our findings also confirmed a dual role of H2A.Z in transcriptional regulation^7^, as the incorporation of either FLAG-HTA9_N or FLAG-HTA9_RR into chromatin was able to restore or partially restore WT expression levels of most of the genes downregulated in *hta9hta11*. In other organism, H2A.Z acetylation at +1 nucleosome region has been shown to play an activating role in transcription^42^. Interestingly, a recent report showed that the two Arabidopsis homologs of yeast Yaf9 are required to activate *FLC* expression through H2A.Z acetylation^41^. In yeast, Yaf9 is a subunit shared by SWR1 and NuA4 complexes, which mediate the deposition of H2A.Z and the acetylation of histone H4, H2A, and H2A.Z, respectively^43^. Loss of YAF9A and YAF9B function causes reduced levels of H2A.Z acetylation at +1 nucleosome region of *FLC*, while H2A.Z deposition is not altered^41^. According to this, H2A.Z acetylation seems to be required for gene activation in Arabidopsis. H2A.Z can be acetylated at several N-terminal lysines^42^. We found that both *hta9hta11/FLAG-HTA9_N* and *hta9hta11/FLAG-HTA9_RR* displayed partially rescued expression levels of many of the downregulated genes in *hta9hta11*. It might be possible that the FLAG-tag at the N-terminal hinders the action of the acetylation machinery. An intriguing possibility could be that the dual role of H2A.Z in regulating gene expression depends on the type of modification that it carries at +1 nucleosome region.

Our data in addition indicated that the transcriptional repressive effect of H2A.Z is not dependent on H3K27me3 as most of the upregulated genes in *hta9hta11* were not marked with H3K27me3 in WT and loss of PRC2 activity did not lead to the upregulation of the same subset of genes than the loss of H2A.Z. Previous reports have showed that the SWR1C mutants *pie1* and *arp6* displayed genome-wide altered levels of H3K27me3^14,39^, suggesting that H2A.Z has a role in promoting H3K27me3 marking. This is in agreement with several data obtained in animal embryonic stem cells when analyzing H3K27me3 levels in H2A.Z knockdown mutants^38,44^. Furthermore, H2A.Zub has been proposed to participate in maintaining H3K27me3 levels at bivalent chromatin in embryonic stem cells^19^. However, H2A.Z and H3K27me3 are enriched at a distinct set of genes in differentiated cells^18,44^, therefore, it is not likely that H2A.Z affects H3K27me3 levels after differentiation. We did not find significant changes in the global levels of H3K27me3 in *hta9hta11* compared to WT or at selected genes; however, our results cannot rule out a possible role of H2A.Z in modulating H3K27me3 deposition at other loci. Nonetheless, it is worth noticing that *pie1* mutants do not fully phenocopy seedlings severely depleted in H2A.Z^2,5^, indicating that PIE1 have additional roles beyond H2A.Z deposition^2,5^ that might affect H3K27me3 levels; In addition, *arp6* displayed altered levels of H3K27me3 and H3K4me3 and altered nucleosome occupancy^14^, indicating that the loss of H2A.Z broadly impact chromatin organization.

On the other hand, loss of CURLY LEAF (CLF) function, which is one of the PRC2 H3K27 trimethyltransferases in Arabidopsis^45^, led to strongly reduced levels of H2A.Z; however, this decrease was not correlated to the loss of H3K27me3, as it occurred even at genes that were not marked with H3K27me3^39^. According to all these data, it might be possible that these chromatin marks reinforce each other effect when co-localizing, but also that the altered levels of one mark indirectly impact the levels of the other mark, especially when the transcriptional activity of the gene change.

In any case, while the number of genes commonly upregulated in loss of H2A.Z and PRC1 mutants clearly suggests a direct connection between H2A.Z regulation and PRC1 activity, the small number of commonly upregulated genes in *hta9hta11* or *pie1* and *fie* or *clf* mutants (our data and ref. ^39^) suggests that H3K27me3, although may have a reinforcing effect, is dispensable for H2A.Z-mediated repression. In agreement with this, a recent report showed that, despite the transcriptional activation of anthocyanin biosynthesis genes in loss of H2A.Z mutants is associated with reduced levels of H2A.Z and H3K27me3, the reduced levels of H3K27me3 are not associated with changes in the expression of these genes^46^, supporting a central role of H2A.Z in the transcriptional repression of these genes.

Interestingly, despite FLAG-HTA9_N and FLAG-HTA9_RR being similarly incorporated into *hta9hta11* chromatin, the transcriptional repressive effect of H2A.Z was significantly recovered only when FLAG-HTA9_N was incorporated, supporting that H2A.Z monoubiquitination, more than H2A.Z incorporation, is what is decisive for H2A.Z-mediated repression. In addition, our results support that the different levels of H2A.Z found at repressed and activated genes are consequence of the transcriptional activity, which is in agreement with recent results in animals^47^. Nonetheless, besides transcriptional regulation, H2A.Z is involved in other processes such as DNA repair and somatic homologous recombination^8^, thus it might be possible that the incorporation and/or distribution of this histone variant follow different rules depending on the process in which is involved.

In summary, based on our and other recent findings we propose a model in which H2A.Z monoubiquitination plays a crucial role in transcriptional repression and, most probably, H2A.Z acetylation in transcriptional activation, while the different levels of H2A.Z along the genes may be a consequence of gene activity rather than an active mechanism to establish repression (Fig. 6d).

## Methods

### Plant material and culture conditions

*Arabidopsis thaliana* Col-0 wild type (WT), *hta9–1hta11–2* (*hta9hta11*)^2^, *atbmi1a/b/c*^25^, *atbmi1a/b* weak^21^ mutants and transgenic plants harboring the different constructs were grown under long day conditions (16 h light and 8 h dark) at 21 °C on MS agar plates containing 1.5% sucrose and 0.8% agar for 7 days. MS agar plates were supplemented with hygromycin (20 µg ml^−1^) for selection of transgenic plants.

### Transgenic plants

*Arabidopsis* native (*N*) or mutated *HTA9* cDNA, in which K129 or both K129 and K132 were replaced by arginine (R), (*RK* and *RR*, respectively), was N-terminally epitope tagged with FLAG and introduced into a modified pCAMBIA 1302 vector (pCAMBIA 130 N)^48^ under the control of the *CaMV35s* promoter. For the incorporation of the FLAG-tag and C-terminal mutations, we designed specific primers that are indicated in Supplementary Table 1. The constructs were introduced into *Agrobacterium* strain GV3101 by the freeze–thaw method^49^ and subsequently transformed into WT, *hta9hta11*, and *atbmi1a/b/c+/*− mutant plants by the floral-dip method^50^.

### Gene expression analysis

Total RNA was extracted from 10 seedlings at 12 DAG using the RNeasy Plant Mini Kit (Qiagen) and on-column DNase treatment (Qiagen) was performed to remove any DNA contamination. cDNAs were reverse-transcribed from total RNAs with the QuantiTect Reverse Transcription Kit (Qiagen). For reverse transcription–PCR analyses, *ACTIN2* was used as endogenous control. Primers used are specified in Supplementary Table 1.

### Protein immunoprecipitation (IP)

One gram of WT, *hta9hta11*/*FLAG-HTA9_N*, or *hta9hta11*/*FLAG-HTA9_RR* seedlings at 7 DAG was collected and fixed in 1% Formaldehyde. Fixed chromatin was extracted and fragmented using a Bioruptor® Pico (Diagenode) in fragments of 200–500 bp. Disrupted chromatin was resuspended in IP buffer (20 mM Tris–HCl pH 8, 150 mM NaCl, 2.5 mM EDTA, 0.5% Triton X-100, and Roche protease inhibitors) and incubated with anti-H3 antibody (abcam, ab1791, 1:300) or anti-HTA9 antibody (agrisera AS10 718, 1:100) at 4 °C overnight with gentle rocking. Protein A magnetic beads (Diagenode kch-802–660) were added and incubated for 4 h at 4 °C with gentle rocking to collect immunocomplexes. After 3–4 washes with IP buffer, immunocomplexes were eluted in Laemmli buffer and boiled for 10 min to analyze by WB.

### Histone extraction

Nuclei from 1 g of 7 day-old seedlings were extracted and treated overnight with 0.4 N H_2_SO_4_ to obtain a histone-enriched extract. The extracted proteins were precipitated with 20% trichloroacetic acid and then washed 3 times with acetone, air-dried, and re-suspended in urea 3 M. Laemmli buffer was added to the samples and boiled for 10 min to analyze by WB.

### Western blot

Proteins were separated on 15% sodium dodecyl sulfate-polyacrylamide gel electrophoresis gel and transferred to a polyvinylidene difluoride membrane (Immobilon-P Transfer membrane, Millipore) by semi-dry blotting in 25 mM Tris–HCl, 192 mM glycine, and 10% methanol. The following primary antibodies were used: anti-HTA9 (agrisera AS10 718, 1:1000), anti-H4 (Abcam ab10158, 1:1000), anti-H3K27me3 (Diagenode, C15410069, 1:1000) anti-AtH2AK121ub (generated in the laboratory^25^, 1:2000), and anti-ubiquitin (Santa Cruz sc-8017, 1:1000); horseradish peroxidase-conjugated goat anti-rabbit (Sigma-Aldrich, A0545) and rabbit anti-mouse (Sigma A9044) were used as secondary antibodies at 1/10,000 and 1/20,000 dilutions, respectively. Chemiluminescence detection was performed with ECL Prime Western Blotting Detection Reagent (GE Healthcare Life Sciences) following the manufacturer’s instructions.

### RNA-seq and RNA-seq data analysis

RNA-seq was performed in two biological replicates for WT, *atbmi1a/b* weak, *hta9hta11*, *hta9hta11/FLAG-HTA9_N*, and *hta9hta11/FLAG-HTA9_RR* at 7 DAG. The Qiagen RNAeasy Mini Kit was used for RNA extraction following the manufacturer’s instructions. RNA concentration and purity were tested using nanodrop photometric quantification (Thermo Scientific). The TruSeq RNA Sample Prep Kit v2 Illumina was used for library preparation following the manufacturer’s recommendations. Sequencing of RNA libraries was carried out with the Illumina HiSeq 2500 sequencer, yielding an average of approximately 35–40 million 100-nucleotide-long paired-end reads for each sample. The high quality of each sample was verified using the software package FASTQC. Read mapping to the *A. thaliana* TAIR10 reference genome and transcript assembly were performed with the software tools HISAT2 and StringTie^51^. The number of genes scored as present in at least one of our samples was 23,486, representing around 70% of Arabidopsis nuclear genes. DEGs were selected using the Bioconductor R packages Ballgown^51^ and LIMMA^52^. Gene expression was measured in FPKM (fragments per kilobase of exon and million mapped reads). DEGs were selected according to a log2-fold change cut-off >|1| in the different comparisons and a *p* value <0.05 according to a moderated *t* test.

### ChIP-seq data analysis

ChIP-seq data for the localization of H3K27me3 and H2AK121ub in WT seedlings at 7 DAG were generated and analyzed previously (accession number GSE89358)^31^. For H2A.Z localization in WT seedlings at 10 DAG, we used previously published data (accession number GSE96834)^32^.

Read mapping to the TAIR10 reference genome was performed using bowtie^53^ and peak calling was carried out with the software package MACS2^54^. All nuclear genes were included in the analysis. Metagene plots were generated with a custom R script based on the R Bioconductor package ChIPeakAnno^55^ and *A thaliana* genome database R package, TxDb.Athaliana.Biomart.plantsmart28^56^. Processing of the BAM files were performed using the software package BEDTools^57^ to obtain RPKM (reads per kilo base per million mapped reads) normalized data.

### ChIP-qPCR

ChIP experiments were performed on 1 g of 7-day-old whole seedlings fixed in 1% Formaldehyde. Chromatin was extracted from fixed tissue and fragmented using a Bioruptor® Pico (Diagenode) in fragments of 200–500 bp. The sheared chromatin was immunoprecipitated overnight using the following antibodies and dilutions: Anti-H3K27me3 (Diagenode, C15410069, 1:300), anti-HTA9 (agrisera AS10 718, 1:100) or anti-H4 (Abcam ab10158, 1:300). Immunocomplexes were captures using Protein A Sepharose beads CL-4B (GE Healthcare). After washing the Protein-A beads, chromatin was eluted and the crosslinking was reversed overnight at 65 °C. The DNA from the immunoprecipitated chromatin was treated with RNase and proteinase K and purified by phenol–chloroform extraction followed by ethanol precipitation. For ChIP-qPCR, amplification was performed using the SensiFAST™ SYBR® & Fluorescein Kit (Bioline) and iQ5 Biorad system. Results are given as the percentage of input normalized to *ACTIN7* or Histone H4. qPCR data are shown as the means of two to four biological replicates. Primers used for ChIP-qPCR are listed in Supplementary Table 1.

### Reporting summary

Further information on research design is available in the Nature Research Reporting Summary linked to this article.

## Supplementary information

Supplementary information

Peer Review

Reporting Summary

Description of Additional Supplementary Files

Supplementary Data 1

Supplementary Data 2

Supplementary Data 3

Supplementary Data 4

Supplementary Data 5

Source Data

## Data Availability

Data supporting the findings of this work are available within the paper and its Supplementary Information files. A reporting summary for this Article is available as a Supplementary Information file. The data sets generated and analyzed during the current study are available from the corresponding author upon request. RNA-seq data sets generated in this study have been deposited in the Gene Expression Omnibus and are available under accession number GSE117969. The source data underlying Figs. 1a, c, e–g, 2a–d, 3e, 5a, c and 6a, c, as well as Supplementary Figs. 1b, 2a, b, 3b and 5a, are provided as a Source Data file.
