# Supplementary material for: The repressive role of Arabidopsis H2A.Z in transcriptional regulation depends on AtBMI1 activity
Source: Nat Commun. 2019 Jun 27;10:2828. doi: 10.1038/s41467-019-10773-1 (PMC6597585; doi:10.1038/s41467-019-10773-1)
Supplement: Supplementary file 1 — Supplementary information [file 41467_2019_10773_MOESM1_ESM.pdf]

The repressive role of Arabidopsis H2A.Z in transcriptional regulation depends on AtBMI1 activity

Gómez-Zambrano *et al.*

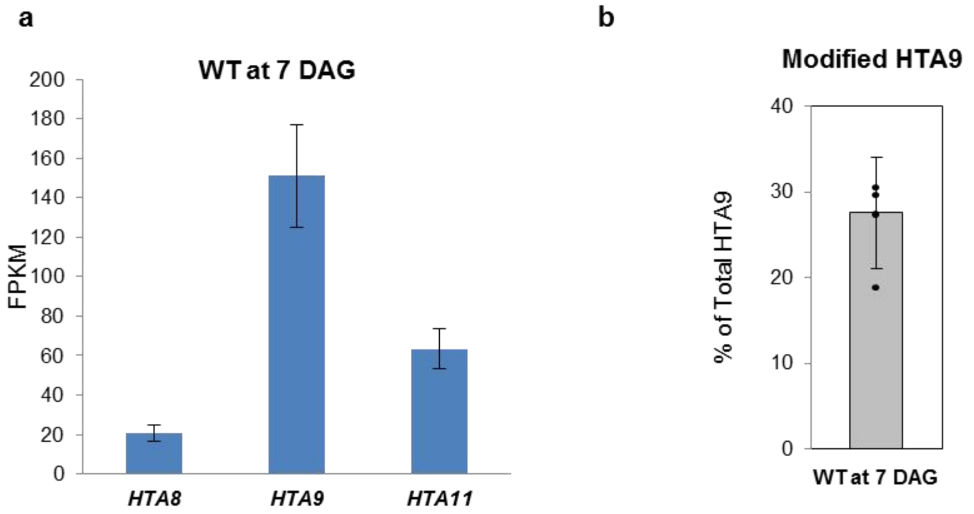

c

```
HTA9_Ath        MSGKGAKGLIMGKPSGSDKDKDKKKPITRSSRAGLQFPVGRVHRLLKTRSTAHGRVGATA
H2A.Z_Human     ---------MAGGKAGKDSGKAKAKAVSRSQRAGLQFPVGRIHRHLKTRTTSHGRVGATA
                         : *  :*.*..* * *.::**.**********:** ****:*:********

HTA9_Ath        AVYTAAILEYLTAEVLELAGNASKDLKVKRISPRHLQLAIRGDEELDTLIKGTIAGGGVI
H2A.Z_Human     AVYSAAILEYLTAEVLELAGNASKDLKVKRITPRHLQLAIRGDEELDSLIKATIAGGGVI
                ***:***************************:***************:***.********

HTA9_Ath        PHIHKSLINKSAKE---
H2A.Z_Human     PHIHKSLIGKKGQQKTA
                ********.*..::
```

Supplementary Figure 1. Arabidopsis H2A.Z coding genes. (a) Expression levels of *HTA8*, *HTA9* and *HTA11* measured in FPKM in WT seedlings at 7 DAG. Error bars indicate standard deviation of n=2 biological replicates. **(b)** Percentage of total HTA9 representing modified HTA9. Error bars indicate standard deviation of n=4 independent WB experiments. Data points were indicated in the bar chart. **(c)** Protein sequence comparison of Arabidopsis HTA9 and human H2A.Z. In red are indicated the possible target lysine/s for monoubiquitination. Source data of Supplementary Figure 1b are provided as a Source Data file.

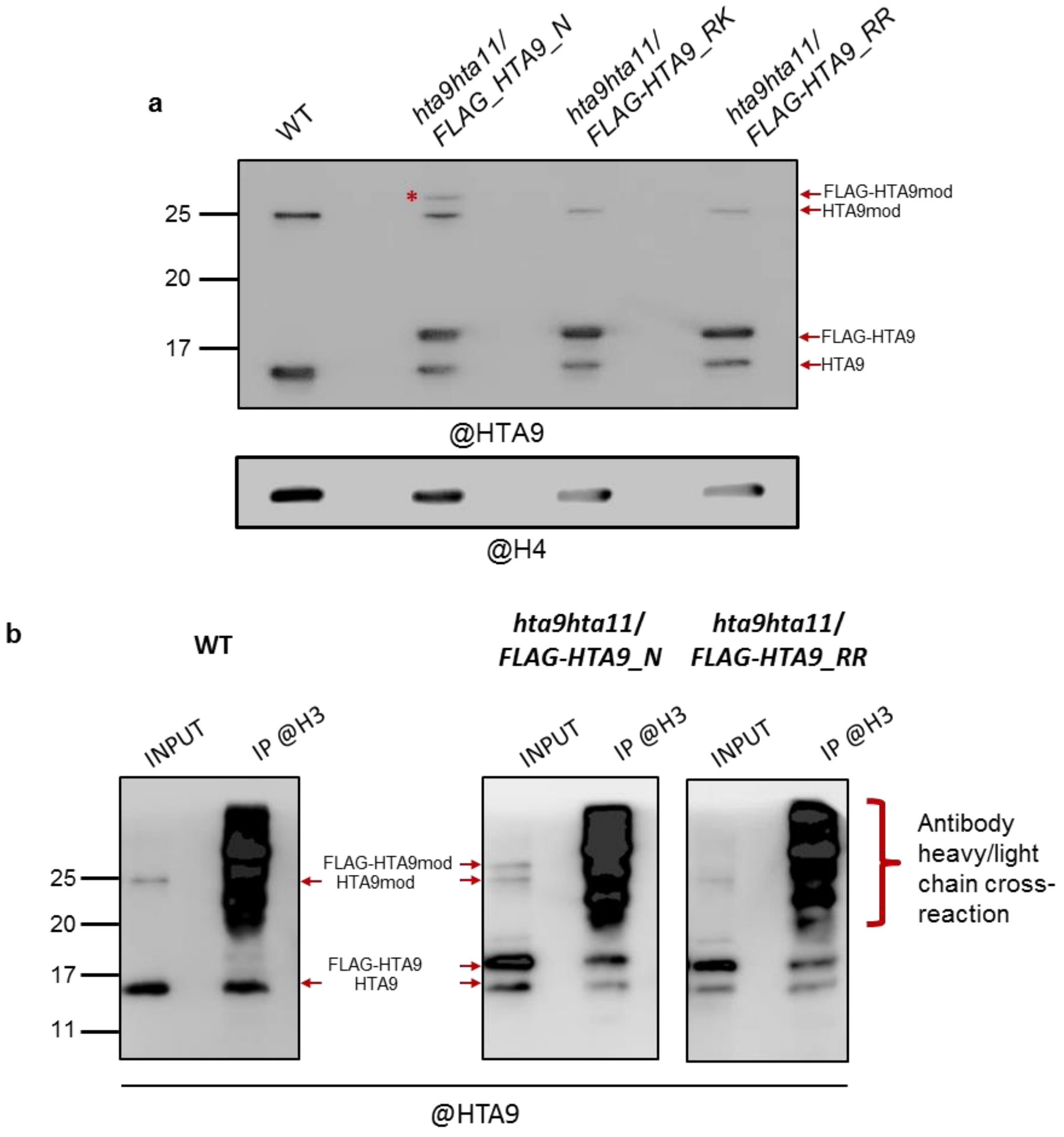

Supplementary Figure 2. Detection of HTA9 and FLAG-HTA9 forms in *hta9hta11/FLAG-HTA9_N, hta9hta11/FLAG-HTA9_RK* and *hta9hta11/FLAG-HTA9_RR* plants. **(a)** WB analysis of HTA9 and FLAG-HTA9 levels in WT, *hta9hta11/FLAG-HTA9_N*, *hta9hta11/FLAG-HTA9_RK* and *hta9hta11/FLAG-HTA9_RR*. The modified FLAG-HTA9 band is only detected in *hta9hta11/FLAG-HTA9_N* plants (asterisk). Some levels of HTA9 and modified HTA9 can be observed in mutant backgrounds as *hta9* is a knockdown allele. **(b)** Detection of HTA9 and FLAG-HTA9 in WT, *hta9hta11/FLAG-HTA9_N* and *hta9hta11/FLAG-HTA9_RR* chromatin immunoprecipitated with anti-H3 antibody. Arrows indicate the bands recognized by the antibody. Source data of Supplementary Figures 2a and 2b are provided as a Source Data file.

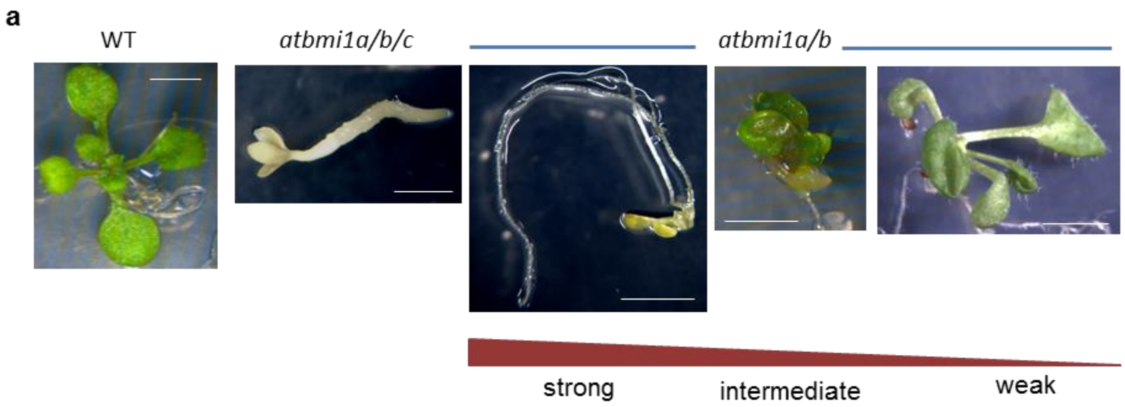

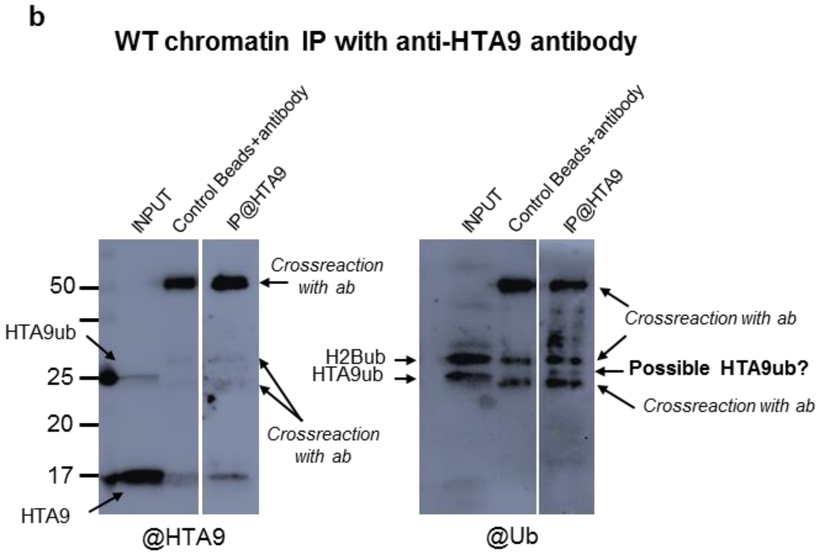

Supplementary Figure 3. Loss of AtBMI1 function mutants and immunoprecipitation of possible HTA9ub. **(a)** *atbmi1a/b/c* mutants are stuck in an embryo maturating phase after germination, while *atbmi1a/b* double mutants display a wide range of phenotypes, showing strong to weak alterations after germination, the weak phenotypes can grow on soil and produce seeds[1]. Bars indicate 2 mm. **(b)** Detection of HTA9ub using anti-ubiquitin antibodies in WT chromatin immunoprecipitated with anti-HTA9 (right panel). A similar blot was probed with anti-HTA9 to verify the presence of HTA9ub and the position of the band (left panel). Source data of Supplementary Figure 3b are provided as a Source Data file.

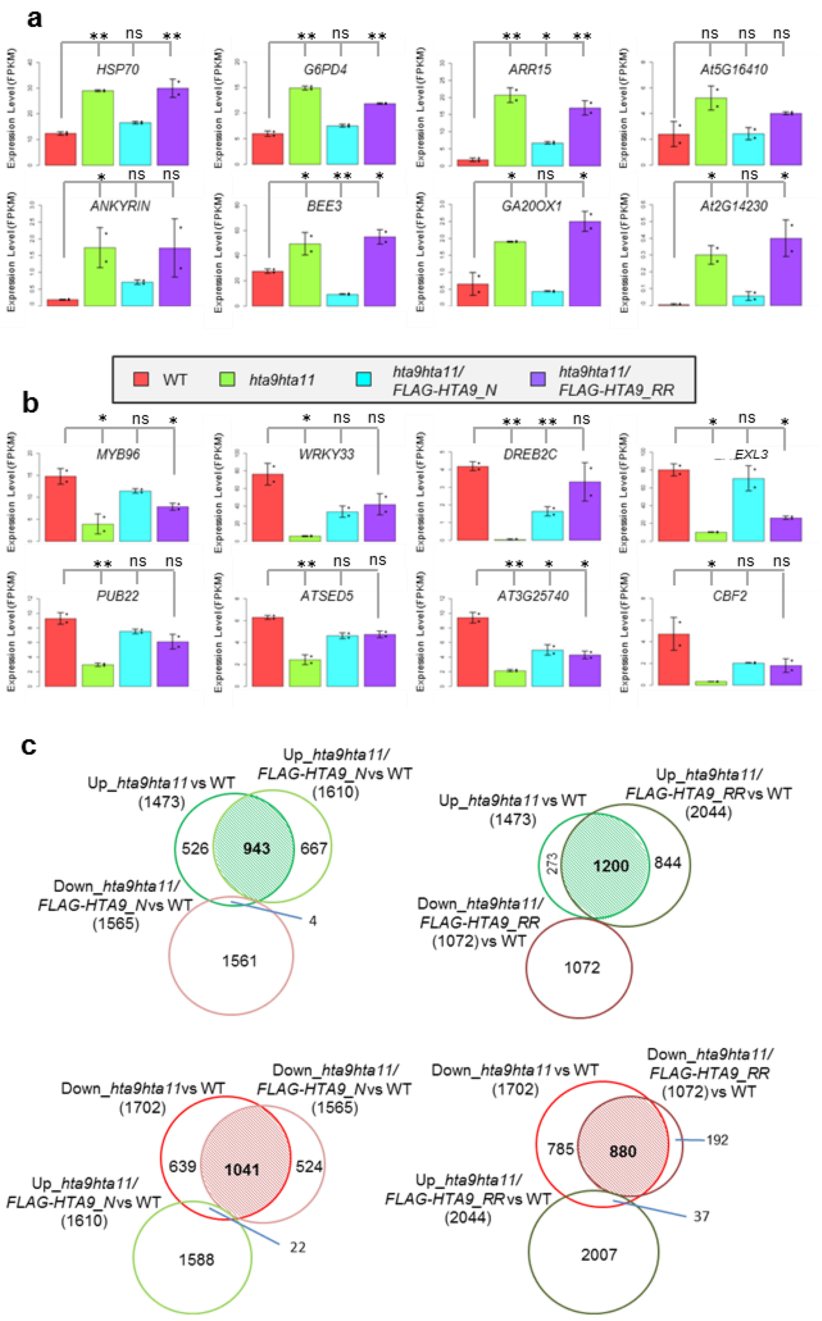

Supplementary Figure 4. Expression levels of several misregulated genes in *hta9hta11* in different genotypes. (a) Expression levels in WT, *hta9hta11*, *hta9hta11/FLAG-HTA9_N* and *hta9hta11/FLAG-HTA9_RR* of representative upregulated genes in *hta9hta11* at 7 DAG determined by RNA-seq. **(b)** Expression levels in WT, *hta9hta11*, *hta9hta11/FLAG-HTA9_N* and *hta9hta11/FLAG-HTA9_RR* of representative downregulated genes in *hta9hta11* at 7 DAG determined by RNA-seq. Expression levels are indicated in FPKM. Error bars indicate standard deviation of n=2 biological replicates. Dots indicate the expression levels in each biological replicate. Significant differences as determined by Student's t-test are indicated (**P < 0.01; *P < 0.05; ns, not significant). **(c)** Genes commonly up (green, two upper panels) and downregulated (red, two bottom panels) in *hta9hta11* and *hta9hta11/FLAG-HTA9_N* or *hta9hta11/FLAG-HTA9_RR*.

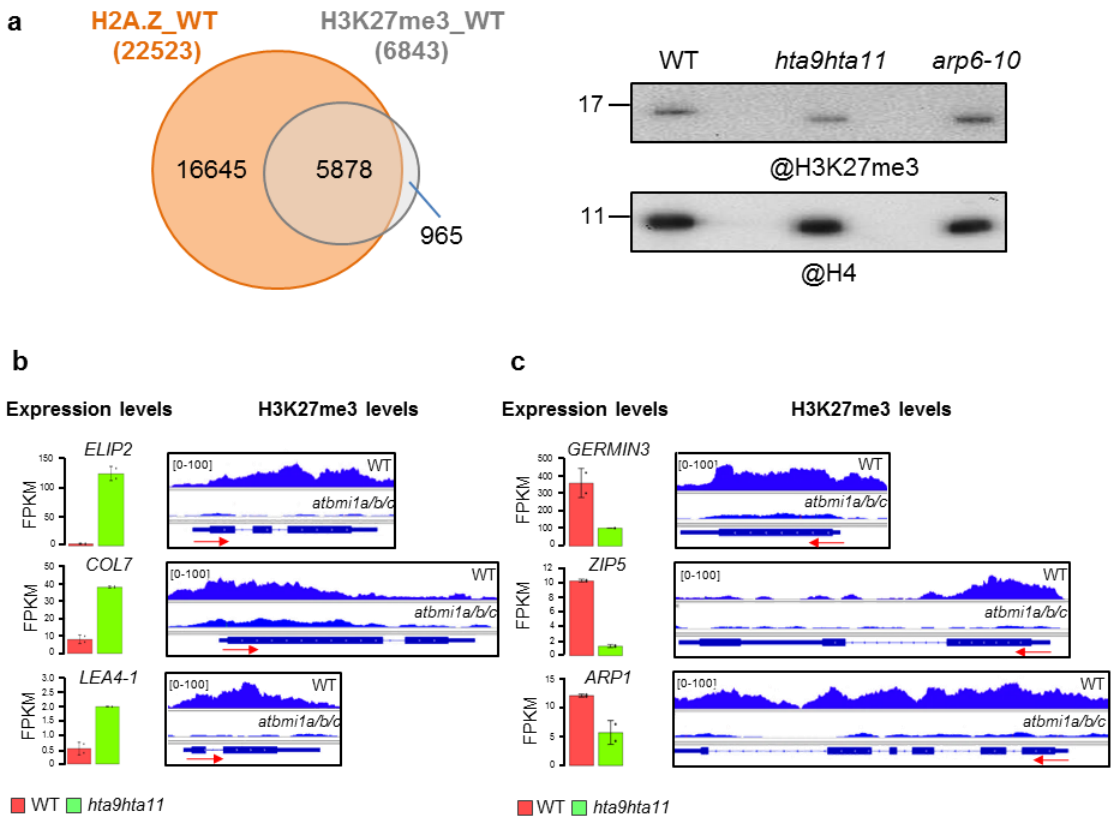

Supplementary Figure 5. Representative *hta9hta11* misregulated genes marked with H3K27me3 in WT that loss the marks in *atbmi1a/b/c* mutants. (a) Left panel, Venn diagram showing overlap between H2A.Z enriched genes and H3K27me3 marked genes in WT seedlings. 85.9% of H3K27me3 marked genes are enriched in H2A.Z. Right panel, Global levels of H3K27me3 in WT, *hta9hta11* and *arp6-10*. **(b)** Expression levels of three upregulated genes in *hta9hta11* indicated in FPKM and ChIP-seq genome browser view of H3K27me3 levels at the genes in WT and *atbmi1a/b/c* mutants. **(c)** Expression levels of three downregulated genes in *hta9hta11* indicated in FPKM (and ChIP-seq genome browser view of H3K27me3 levels at the genes in WT and *atbmi1a/b/c* mutants. Dots indicate the expression levels in each biological replicate. Error bars indicate standard deviation of n=2 biological replicates. Source data of Supplementary Figure 5a are provided as a Source Data file.

a

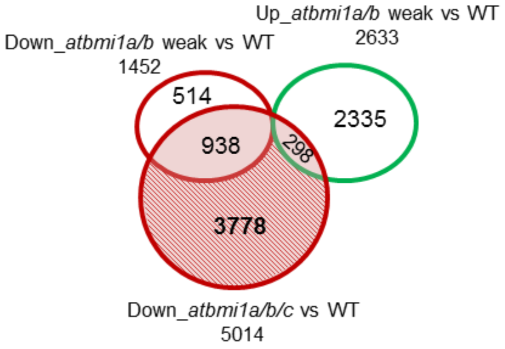

b

Gene	*Genotype*	Expression levels (FPKM)
FBA2	WT	1433,3
	atbmi1a/b weak	1479,3
	atbmi1a/b/c	148,1
LHCB2	WT	1813,0
	atbmi1a/b weak	1748,5
	atbmi1a/b/c	34,4

c

Gene	*Genotype*	Expression levels (FPKM)
HSP70	WT	12,3
	atbmi1a/b weak	101,8
	atbmi1a/b/c	374,0
ELIP2	WT	2,9
	atbmi1a/b weak	224,1
	atbmi1a/b/c	255,0

Supplementary Figure 6. The levels of H2A.Z are higher at transcriptionally repressed genes. (a) Venn diagram showing the number of genes downregulated in *atbmi1a/b/c* but not in *atbmi1a/b weak*. **(b)** Gene expression levels of *FBA2* and *LHCB2* indicated in FPKM, which are genes downregulated in *atbmi1a/b/c* but not in WT *or atbmi1a/b* weak. **(c)** Gene expression levels of *HSP70* and *ELIP2* indicated in FPKM, which are upregulated in both *atbmi1a/b* weak and *atbmi1a/b/c* mutants.

Supplementary Table 1. Primers used in this work.

FLAG-HTA9 Fw	AAACCATGGATTACAAGGATGACGACGATAAGATGTCGGGGAAAGGT
HTA9-N Rev	TTTGGATCCCTATTCCTTGGCGGATTTGTTGAT
HTA9-RK Rev	TTTGGATCCCTATTCCTTGGCGGATCGGTTGAT
HTA9-RR Rev	TTTGGATCCCTATTCCCGGGCGGATCGGTTGAT
qRT-PCR-FT Fw	CGAACGGTGATGATGCCTATAGTAG
qRT-PCR-FT Rev	CACTCTCATTTTCCTCCCCCTCTC
qRT-PCR-FLCFw	GCCACCTTAAATCGGCGGTTG
qRT-PCR-FT Rev	CACAAAGTCTCTTGGCCAAAGAGAGAG
qRT-PCR-ACT2 Fw	GTAACATTGTGCTCAGTGGTGG
qRT-PCR-ACT2 Rev	CTCGGCCTTGGAGATCCACATC
qPCR-ELIP2 1Fw	TCGTTTAACATGCAGTCAGT
qPCR-ELIP2 1Rev	GAGGAGACTGTGGCATTT
qPCR-ELIP2 2Fw	GGTGTCGGGTGGTTTCTAGG
qPCR-ELIP2 2Rev	CATAGCTCGGCGTCTGAAGT
qPCR-LEA4-1 Fw	AGCCAAGGAGAAGATGGTGA
qPCR-LEA4-1 Rev	CGTTGGTGCGCTATCTCTTT
qPCR-COL7 Fw	GTTCTTCTCAAGGCCTCTCT
qPCR-COL7 Rev	CCAACTAGCACGTGATCTCT
qPCR-FBA2 1Fw	TTCCGGACTTTGCTGGTCTC
qPCR-FBA2 1Rev	ATGTTCTGCTCGACGAGGAC
qPCR-FBA2 2Fw	TTCCGGACTTTGCTGGTCTC
qPCR-FBA2 2Rev	ATGTTCTGCTCGACGAGGAC
qPCR-GER3 Fw	CTTTTGCCTCTGTTCAAGAC
qPCR-GER3 Rev	GGCTTTAATGATGTTGGAAG
qPCR LHCB2 1Fw	GAAGCAATGGCCACTTCAGC
qPCR LHCB2 1Rev	CGGAGGAGATCGTTGGATGG
qPCR LHCB2 2Fw	ACCCGTTGAACTTAGCGGAG
qPCR LHCB2 2Rev	TAGCCACAGGGTCTGCAATG
qPCR-ARP1 Fw	GTCCACCAATCTGTCACTCT
qPCR-ARP1 Rev	CTCCTACGAACACTTTGGTC
qPCR-ZIP5 Fw	CACAAAACGTCAAGCTCTTA
qPCR-ZIP5 Rev	TTGCCTAGTAACGGAAACAT
qPCR-ACT7 Fw	GCGATGTTTGAGTTTCAATAAACGCTGC
qPCR-ACT7 Rev	CTCACCTTCACCATTCCAGTTCCA
qPCR-HSP70 1Fw	TCTCTTAAAGCTCACAGACGAA[2]
qPCR-HSP70 1Rev	CTGTCAGTGAAAGCAACGTAGG[2]
qPCR-HSP70 2Fw	GTCGAGTGTTGGCGAGAAGA
qPCR--HSP70 2Rev	CCGCTCACAAGCTGTCCTAA
qPCR-FLC 1Fw	CTCCTCCGGCGATAAGTACG
qPCR-FLC 1Rev	GCTGACGAGCTTTCTCGATG
qPCR-FLC 2Fw	TCTGGTTATCGATTGCGATTCT
qPCR-FLC 2Rev	GTTCTCTTGGATTTGTATATGCACG

Supplementary References

1. Bratzel, F., López-Torrejón, G., Koch, M., Del Pozo, J. C. & Calonje, M. Keeping cell identity in Arabidopsis requires PRC1 RING-finger homologs that catalyze H2A monoubiquitination. *Curr. Biol.* **20**, 1853–1859 (2010).

2. Kumar, S. V. & Wigge, P. A. H2A.Z-containing nucleosomes mediate the thermosensory response in Arabidopsis. *Cell* **140**, 136–147 (2010).
