# Supplementary material for: The repressive role of Arabidopsis H2A.Z in transcriptional regulation depends on AtBMI1 activity
Source: Nat Commun. 2019 Jun 27;10:2828. doi: 10.1038/s41467-019-10773-1 (PMC6597585; doi:10.1038/s41467-019-10773-1)
Supplement: Supplementary file 3 — Reporting Summary [file 41467_2019_10773_MOESM3_ESM.pdf]

Corresponding author(s): Myriam Calonje

Reporting Summary

Nature Research wishes to improve the reproducibility of the work that we publish. This form provides structure for consistency and transparency in reporting. For further information on Nature Research policies, see Authors & Referees and the Editorial Policy Checklist.

Statistical parameters

When statistical analyses are reported, confirm that the following items are present in the relevant location (e.g. figure legend, table legend, main text, or Methods section).

n/a	Confirmed	
☐	☒	The exact sample size (*n*) for each experimental group/condition, given as a discrete number and unit of measurement
☐	☒	An indication of whether measurements were taken from distinct samples or whether the same sample was measured repeatedly
☐	☒	The statistical test(s) used AND whether they are one- or two-sided<br>*Only common tests should be described solely by name; describe more complex techniques in the Methods section.*
☒	☐	A description of all covariates tested
☐	☒	A description of any assumptions or corrections, such as tests of normality and adjustment for multiple comparisons
☐	☒	A full description of the statistics including central tendency (e.g. means) or other basic estimates (e.g. regression coefficient) AND variation (e.g. standard deviation) or associated estimates of uncertainty (e.g. confidence intervals)
☐	☒	For null hypothesis testing, the test statistic (e.g. *F*, *t*, *r*) with confidence intervals, effect sizes, degrees of freedom and *P* value noted<br>*Give P values as exact values whenever suitable.*
☒	☐	For Bayesian analysis, information on the choice of priors and Markov chain Monte Carlo settings
☐	☒	For hierarchical and complex designs, identification of the appropriate level for tests and full reporting of outcomes
☒	☐	Estimates of effect sizes (e.g. Cohen's *d*, Pearson's *r*), indicating how they were calculated
☐	☒	Clearly defined error bars<br>*State explicitly what error bars represent (e.g. SD, SE, CI)*

Our web collection on statistics for biologists may be useful.

Software and code

Policy information about availability of computer code

Data collection	H2A.Z ChIP seq data were collected from Gene Expression Omnibus (GEO) with accession number GSE96834 (Wollmann, H. et al., 2017) and H3K27me3 and H2AK121ub ChIP seq data form GEO under accession GSE89358 (Zhou, Romero-Campero et al., 2017)
Data analysis	Sequencing of RNA libraries was carried out with the Illumina HiSeq 2500 sequencer. The high quality of each sample was verified using the software package FASTQC. Read mapping to the A. thaliana TAIR10 reference genome and transcript assembly were performed with the software tools HISAT2 and StringTie. Differentially expressed genes (DEGs) were selected using the Bioconductor R packages Ballgown and LIMMA. Gene expression was measured in FPKM (fragments per kilobase of exon and million mapped reads). Differentially expressed genes were selected according to a log2-fold change cut-off>|1| in the different comparisons and a pvalue <0.05.<br>ChIP-seq data for the localization of H3K27me3 in WT seedlings at 7 DAG were generated and analyzed previously (Zhou, Romero-Campero et al., 2017). For H2A.Z localization in WT seedlings at 10 DAG we used previously published data (Wollmann et al. 2017); accesion number GSE96834). Read mapping to the TAIR10 reference genome was performed using bowtie and peak calling was carried out with the software package MACS2. Metagene plots were generated with a custom R script based on the R Bioconductor packages ChIPeakAnno and Arabidopsis thaliana genome database R package, TxDb.Athaliana.Biomart.plantsmart2843. Processing of the BAM files were performed using the software package BEDTools to obtain RPKM normalized data. The area under the curve (AUC) was calculated using RPKM values at the defined regions of the gene (from TSS to 200 bp downstream TSS for +1 nucleosome region and from TSS to TES for entire gene length) with the zoo R package. H2A.Z signal was estimated by summing AUC of each gene.

For manuscripts utilizing custom algorithms or software that are central to the research but not yet described in published literature, software must be made available to editors/reviewers upon request. We strongly encourage code deposition in a community repository (e.g. GitHub). See the Nature Research guidelines for submitting code & software for further information.

Data

Policy information about availability of data

All manuscripts must include a data availability statement. This statement should provide the following information, where applicable:

- Accession codes, unique identifiers, or web links for publicly available datasets
- A list of figures that have associated raw data
- A description of any restrictions on data availability

All materials from this study are freely available from the authors. The RNA-seq datasets generated in this study have been deposited in the Gene Expression Omnibus (GEO) under accession GSE117969.

Field-specific reporting

Please select the best fit for your research. If you are not sure, read the appropriate sections before making your selection.

☒ Life sciences ☐ Behavioural & social sciences ☐ Ecological, evolutionary & environmental sciences

For a reference copy of the document with all sections, see nature.com/authors/policies/ReportingSummary-flat.pdf

Life sciences study design

All studies must disclose on these points even when the disclosure is negative.

Sample size	For flowering time analyses, we used at least 20 plants of each genotype.<br>For qRT-PCR analyses, we used n=4 biological replicates of each genotype. For each replicate RNA was extracted from 10 seedlings at 12 DAG.<br>For Western blot quantifications, we used n=3 to 4 biological replicates of each genotype. For each replicate, Histone enriched extracts were obtained from 1 gr of seedlings at 7 DAG.<br>For RNA-seq analyses, we used n=2 biological replicates of each genotype. For each replicate 5 mgr. of RNA was used for the analysis.<br>For ChIP-qPCR analyses, we used n=2 to 4 biological replicates of each genotype. For each replicate chromatin was extracted from 1 gr. of seedlings at 7 DAG.
Data exclusions	We did not apply Data exclusion.
Replication	Experimental findings were reliably reproduced as indicated by the error bars of independent biological replicates.
Randomization	A. thaliana plants of different genotype were randomized across 1-2 trays in growth chamber conditions.
Blinding	We did not apply blinding group allocation during sampling.

Reporting for specific materials, systems and methods

Materials & experimental systems

n/a	Involved in the study
☒	☐ Unique biological materials
☐	☒ Antibodies
☐	☒ Eukaryotic cell lines
☒	☐ Palaeontology
☒	☐ Animals and other organisms
☒	☐ Human research participants

Methods

n/a	Involved in the study
☒	☐ ChIP-seq
☒	☐ Flow cytometry
☒	☐ MRI-based neuroimaging

Antibodies

Antibodies used	The following primary antibodies were used: anti-HTA9 (agrisera AS10 718), anti-H4 (Abcam ab10158), anti-H3K27me3 (Diagenode, C15410069) anti-AtH2AK121ub (generated in the laboratory (Yang et al., 2013); anti-ubiqitin (Santa Cruz sc-8017) and anti-H3 antibody (abcam, ab1791)
Validation	anti-HTA9 (agrisera AS10 718), specific for Arabidopsis HTA9 (https://www.agrisera.com/en/artiklar/hta9-probable-histone-h2a-variant-3.html)<br>anti-H4 (Abcam ab10158), Validated for ChIP in Arabidopsis (Pacinka et al., 2010).

anti-H3K27me3 (Diagenode, C15410069), Validated for WB and ChIP in Arabidopsis (https://www.diagenode.com/en/p/h3k27me3-polyclonal-antibody-classic-50-mg-34-ml).
anti-AtH2AK121ub, generated and validated for WB in the laboratory (Yang et al., 2013)
anti-ubiqitin (Santa Cruz sc-8017), validated for Arabidopsis in the laboratory (Bratzel et al., 2010).
anti-H3 antibody (abcam, ab1791), validated for Arabidopsis (https://www.abcam.com/histone-h3-antibody-nuclear-loading-control-and-chip-grade-ab1791.html?productWallTab=Abreviews).

Eukaryotic cell lines

Policy information about cell lines

Cell line source(s)	We did not use eukaryotic cell lines in our study. We used Arabidopsis TDNA insertion lines which were obtained from public stock centers and previously characterized or generated new TDNA lines by the floral-dip method for this work.
Authentication	We confirmed the T-DNA insertion lines by locus specific PCR using genomic DNA as template, by gene expression and/or protein expression.
Mycoplasma contamination	Not applicable
Commonly misidentified lines (See ICLAC register)	Not applicable
