# Supplementary material for: The repressive role of Arabidopsis H2A.Z in transcriptional regulation depends on AtBMI1 activity
Source: Nat Commun. 2019 Jun 27;10:2828. doi: 10.1038/s41467-019-10773-1 (PMC6597585; doi:10.1038/s41467-019-10773-1)
Supplement: Supplementary file 4 — Description of Additional Supplementary Files [file 41467_2019_10773_MOESM4_ESM.docx]

**Description of Additional Supplementary Files**

File Name: Supplementary Data 1
Description: H2A.Z enriched, H3K27me3 marked and H2AK121ub marked genes in WT seedlings

File Name: Supplementary Data 2
Description: Misregulated genes in *hta9hta11*, *atbmi1a/b* weak, *hta9hta11/FLAG-HTA9_N* and *hta9hta11/FLAG-HTA9_RR* compared to WT at 7 DAG

File Name: Supplementary Data 3
Description: Misregulated genes in *hta9hta11/FLAG-HTA9_N* and *hta9hta11/FLAG-HTA9_RR* compared to *hta9hta11* at 7 DAG

File Name: Supplementary Data 4
Description: Expression levels of the genes commonly misregulated in *hta9hta11* and *hta9hta11/FLAG-HTA9_N* or *hta9hta11/FLAG-HTA9_RR*

File Name: Supplementary Data 5
Description: Misregulated genes in *atbmi1a/b/c* compared to WT at 7 DAG
