# Supplementary material for: The repressive role of Arabidopsis H2A.Z in transcriptional regulation depends on AtBMI1 activity
Source: Nat Commun. 2019 Jun 27;10:2828. doi: 10.1038/s41467-019-10773-1 (PMC6597585; doi:10.1038/s41467-019-10773-1)
Supplement: Supplementary file 2 — Peer Review [file 41467_2019_10773_MOESM2_ESM.pdf]

Reviewers' comments:

Reviewer #1 (Remarks to the Author):

Gomez-Zambrano et al. present evidence that the histone variant H2A.Z is ubiquitinated by the PRC1 complex in Arabidopsis, and that this activity underlies the repressive effects of H2A.Z on transcription. This is an important discovery that helps to clarify long-standing questions of how H2A.Z can serve as both an activator and repressor of transcription. However, some of the conclusions of this manuscript are not well founded and, in my opinion, require additional work or alterations to the authors' interpretations. Below I outline each of my concerns with this paper:

1. The evidence for ubiquitination of HTA9 by BMI is quite strong considering the data presented in Figure 1A and C and Figure 2A-E. However, there remains some level of uncertainty as to whether this modified form of HTA9 is truly a monoubiquitination at K129, as this is not directly demonstrated. While an anti-ubiquitin antibody reacts with a band of the same size as HTA9 in WT and not in atbmi1a/b/c mutants (Figure 2C), it remains uncertain whether this really is HTA9 monoubiquitinated at K129. Other questions arise from this panel that stir doubts. For example, are H2Bub and HTA9ub really the only ubiquitinated species in this histone-enriched extract? Where is H2Aub? In my opinion, the conclusions from this part of the paper would be greatly strengthened by direct analysis of HTA9 modifications by mass spectrometry. At the least, the authors could perform HTA9 IP followed by a western blot for ubiquitin on WT, hta9/hta11, and bmi1a/b/c plants.

2. With respect to complementation of hta9/hta11 mutants with the FLAG-HTA9_N construct, the data suggest that this is not a complete rescue, which confounds some of the subsequent interpretations. For example, the early flowering of hta9/hta11 is known to result from lack of expression of FLC in the absence of H2A.Z deposition. Thus, the fact that hta9/hta11/ FLAG-HTA9_N does not completely restore normal flowering time (Fig 1D and E), suggests that the fusion protein is not fully functional. Consistent with this notion is the fact that many genes that require H2A.Z for activation are not properly expressed in the hta9/hta11/ FLAG-HTA9_N plants (Supplemental Figure 4B). If N-terminal acetylation is important for activation, then this lack of complete complementation may be explained by having an epitope tag at the N-terminus. The authors should at least consider this in their interpretations of the data.

3. Related to Figure 2E, and Page 5 paragraph 2, it is not reasonable to conclude anything about H2AK121ub being able to functionally replace H2A.Zub based on these experiments. We don't know that these histones were chromatin bound or if H2AK121ub and H2A.Zub are even at the same sites. This part of the text needs modification.

4. Based on Figure 4, the authors conclude that H2A.Z and H3K27me3 are essentially independent of one another at a functional level. However, the authors do not discuss recent results that seem to show an interdependence between H2A.Z deposition and H3K27me3 deposition (Carter et al [2018] Plant Cell 30). The apparent contradiction between those results and the present manuscript must be discussed.

5. Regarding Figure 5A, it is very hard to believe that the S. pombe H2A.Zac antibody reacts with H2A.Z from Arabidopsis given the differences in amino acid sequence. Based on the weak bands shown on this western blot, I am not convinced. Further, if it is in fact reacting with acetylated H2A.Z, is it mono-, di-, tri-, or tetracetylated? There are ways to test this more directly, but I am not sure it is worthwhile in the context of this manuscript.

6. Finally, on page 8 the authors conclude that modifications of H2A.Z at the +1 nucleosome are all that matters with respect to transcriptional regulation. I do not see any data that directly support this claim.

Reviewer #2 (Remarks to the Author):

The authors of this manuscript report that H2A.Z can be monoubiquitinated at K129 and that this modification is deposited by PCR1. This modification causes repression, which is independent of PRC2. They furthermore propose that H2A.Z can be acetylated, which likely causes gene activation. H2A.Z monoubiquitination by PRC1 has been shown before in mammals (Sarcinella et al. 2007), but not in plants. Likewise, H2A.Z acetelation has previously been shown in yeast, protozoans, and metazoans (Ren and Gorovsky 2001, Bruce et al. 2005, Babiarz et al. 2006), but not in plants. The link of H2A.Z acetylation with gene activation has been shown in animals (Bruce et al 2005, Vandés-Mora 2012), but not in plants. Therefore, while the discoveries are not entirely novel, they are nevertheless a novelty for plants. The major claims of the manuscript are supported by the data shown; however, I have some comments that remain to be addressed:

1. The authors claim that „ the early flowering phenotype of hta9hta11 was rescued in hta9hta11/FLAG-HTA9_N plants but not in mutants expressing FLAG-HTA9_RK or FLAG-HTA9_RR (Fig. 1d,e), which was supported by FT transcript levels (Fig. 1f)." To make the claim that the phenotype of hta9hta11 was rescued in hta9hta11/FLAG-HTA9_N plants, they should compare the difference between wild-type and hta9hta11/FLAG-HTA9_N; which based on the data will be significant; they can therefore only conclude that there was a partial rescue. In addition, the second claim is not correct, since there is a significant difference between hta9hta11 and FLAG-HTA9_RK or FLAG-HTA9_RR expressing plants; they can therefore only conclude that both constructs do not fully complement the phenotype of the mutant. Furthermore, the FLAG-HTA9_RR line clearly differs from the hta9/11 mutant, as evident by the data shown in Figure 3.

2. Figure 3a: The comparison is highly confusing. Why did the authors not directly compare FLAG-HTA9_N with FLAG-HTA9RR? They should also reverse the order of the comparison and show hta9hta11 versus FLAG-HTA9_N; so that is clear that upregulated genes are upregulated as a consequence of impaired H2A.Z function.

3. A study on mouse ES cells showed that ca. 25% of H2A.Zub is also acetylated (Ku et al. 2012). Another study showed that the difference in acetylation versus non-acetylation explains the difference in effect on transcription (Vandés-Mora 2012). The authors could also use (reciprocal) IP followed by WB to determine whether this double-marking is the case in Arabidopsis. If these modifications turn out to co-occur in the majority of the cases, then the model is not as clear-cut as they suggest.

Other comments
• Page 3, top. Is the indication that H2A.Z is about 20% of H2A.Z based on quantification?
• The authors have an anti-H3 IP (protein immunoprecipitation) method in their methods section, but I cannot see any result obtained with such IP.
• Subjective wording/rephrasing:
-"very similar phenotype" on page 2. Remove the word "very".
-"small fraction", change to minor fraction (as opposed to major)
-Figure 1 f , multiplication signs lacking on Y-axis labels.
-Figure 4b requires more intuitive labeling
-In Figure 4c: are the 3 upregulated and 3 downregulated genes part of the overlapping fields in Figure 4b? This is not immediately clear from the text.
-Page 4: throughout plant developmental
-Page 4: PRC1 acts together PRC2

Reviewer #3 (Remarks to the Author):

The authors identified monoubiquitination of K129 of H2A.Z as a modification important for this histone variant, which can be partially responsible for repressive impact on the transcription. Furthermore, they identified the major enzyme responsible for the modification. The data are convincing and I have no objections to the presentation of this part of the manuscript.

On the other hand, the part about H2A.Z acetylation is undeveloped. The western analysis using anti-phospho Ab shows much higher signal from H4 than from H2A.Z. I understand the authors explanation, but this result is not suitable for publishing – different, more specific Ab should be generated. This part is a bit out of scope of the paper. Since extensive additional data would need to be added for a proper analysis of histone phosphorylation, I would suggest to completely remove this part from the manuscript.

The authors suggest that H2A.Zub occurs at +1 nucleosome, however there is no clear evidence that this is the case. I would be happy to see some ChIP data showing that indeed ubiquitination occurs predominantly at +1 nucleosomes.

The authors generated a really nice tool to study H2A.Z ubiquitination, FLAG-HTA9_RR and FLAG-HTA9_N lines, which is however not fully exploited in the context of the atbmi1 triple mutant. Performing some ChIP analysis of HTA9 for both constructs in the background of the atbmi1 triple mutant would provide information whether H2A.Z ubiquitination is crucial for the removal of H2A.Z from the nucleosomes upon transcriptional activation. I understand, however, that this experiment would require generating multiple mutant lines (hta9/11 atbmi1a/b/c) which needs to carry HTA9 transgenes. Therefore, I leave the decision whether this experiment is required to the competence of the Editors.

Some conclusions in the ms are overstated and do not find confirmation in the data (please see below for details). The model proposed by the authors is interesting however too speculative. First, placing H2A.Zub in the +1 nucleosome is not supported in the data. Second, the role of H2A.Z acetylation in activating gene transcription is not supported at all. I suggest to remove the right-hand part of the model and change H2A.Z ubiquitination based on new data, which should be provided. It would be good to improve images of nucleosomes (especially the DNA wrapping is very weird).

In general, this is an important study as it provides some clues about how H2A.Z histone variant can adopt different, antagonistic roles in regulation of transcription. From this perspective I believe it would be of interest for Nat Comm. readers and I recommend accepting the ms after revision.

Specific comments:
Fig. S2A. How the authors would explain the fact that WT HTA9 show at least 3x higher level of its modified version in FLAG-HTA9-N line that in FLAG-HTA9-RK and -RR lines? I am afraid whether the lack of the modified version of FLAG-HTA9 in those two lines could be due to apparent decreased detection level in the two mutated lines.

p. 4
"We found that the early flowering phenotype of hta9hta11 was rescued in hta9hta11/FLAG-HTA9_N plants but not in mutants expressing FLAG-HTA9_RK or FLAG-HTA9_RR (Fig. 1d, e), which was supported by FT transcript levels (Fig. 1f)."
According to the results shown in Figs. 1d, e, f the phenotype of FLAG-HTA9_RK and _RR lines also seems to be improved. The authors should indicate this, even though it is not statistically significant, as the data for both types of experiments (flowering time and FT expression) and both lines are very consistent.

p. 4
"This activity is required to maintain gene repression throughout plant developmental." Should be "plant development"

p.4
"atbmi1a/b/c mutants remain in an embryo maturating-like phase due to misexpression of the embryonic program after germination25 (Supplementary Fig. 3). Moreover, 20% of Arabidopsis transcriptome is misregulated in these mutants"
This is one triple mutant or several different triple mutants? In the first case, change the plural for singular. Please, change accordingly in the whole ms.

p.4
"In fact, PRC2 to incorporate H3K27me3 requires the presence of H2AK121ub marks at these genes31" change the word order to "In fact, PRC2 requires the presence of H2AK121ub marks to incorporate H3K27me3 at these genes31"

p. 5
"Since HTA9ub represented a small percentage of total HTA9, it was reasonable to assume that it localizes at +1 nucleosome region."
This is a wide speculation, in addition based on wrong assumptions. It is well-known that H2A.Z localizes predominantly at +1 nucleosome; this location constitutes about 80% of total H2A.Z or even more. Therefore, the authors should argue that it resides in gene body rather than in +1, if they want to use the argument of similar percentage, which in general I think is irrational.

p.5 and Fig. 3c and d
"Venn diagrams showing the percentage of genes upregulated in hta9hta11 that are not upregulated in hta9hta11/FLAG-HTA9_N (left panel)"
I understand that the diagrams show up- and down-regulation of genes in the three lines when compared to wt? If this is the case, it should be clearly stated in the figure caption. In addition.

Fig. S4. The way of data presentation suggests RT-qPCR? Only after checking the units on the axis you can find that this is RNA-seq data. This should be made more clear in the figure caption.

p. 8
"(…) since H2A.Z levels at repressed genes are higher than at active genes in WT (Fig. 2f), it might be possible that the increased levels of HTA9 in atbmi1a/b/c are a consequence of the high number of downregulated genes."
I do not understand this conclusion, since the number of upregulated genes in atbmi1 triple is higher than downregulated ones. I would rather speculate that increased amount of HTA9 in the mutant is a way to compensate the lack of ubiquitination since both the H2A.Zub and H2A.Z alone show repressive impact on transcription.

p. 8
"On the other hand, the levels of H2A.Z at genes are a consequence of the transcriptional activity."
I think that this is overstated. I don't see any evidence in the authors' data that changes of H2A.Z level during transcriptional activation and repression are consequences of the transcription (although I think this is likely).

Response to Reviewers

We would like to thank the Reviewers for their comments and suggestions that have helped us to improve the manuscript. We have included new data and discussion to clarify the points raised by Reviewers. We have added a version of the manuscript in which text changes are highlighted in yellow. Below we line out our changes in a point by point reply to the Reviewers comments.

We have also modified our manuscript to comply with NCOMMS format requirements. We have removed references from the Abstract, added a short introduction, included a results section divided by subheadings and a discussion. I hope this will not hinder the revision process by the reviewers.

Response to Reviewer #2

1. The evidence for ubiquitination of HTA9 by BMI is quite strong considering the data presented in Figure 1A and C and Figure 2A-E. However, there remains some level of uncertainty as to whether this modified form of HTA9 is truly a monoubiquitination at K129, as this is not directly demonstrated. While an anti-ubiquitin antibody reacts with a band of the same size as HTA9 in WT and not in atbmi1a/b/c mutants (Figure 2C), it remains uncertain whether this really is HTA9 monoubiquitinated at K129. Other questions arise from this panel that stir doubts. For example, are H2Bub and HTA9ub really the only ubiquitinated species in this histone-enriched extract? Where is H2Aub? In my opinion, the conclusions from this part of the paper would be greatly strengthened by direct analysis of HTA9 modifications by mass spectrometry. At the least, the authors could perform HTA9 IP followed by a western blot for ubiquitin on WT, hta9/hta11, and bmi1a/b/c plants.

We agree with Reviewer that the analysis of HTA9ub by mass spectrometry would confirm that K129 is the predominant target lysine; however, for this analysis it would be required to purify HTA9ub or at least to have a specific anti-HTA9ub antibody to immunoprecipitate HTA9ub before mass spec; unfortunately, the antibody is not yet available. HTA9ub represents only 20% of total HTA9, therefore, if we immunoprecipitate histone extracts with anti-HTA9, we are afraid that most of the immunoprecipitated fraction would be HTA9 and HTA9ub might not be detected. We could also try to cut the band corresponding to HTA9ub from a gel for the analysis; however, histone extracts are enriched in histones but contain many other proteins. The mass of the monoubiquitinated peptide should be predicted from a long list of peptides coming from unknown proteins, thus, this would be only a prediction. We have mutated HTA9 at K129 on one hand and at K129 and K132 on the other one. In both cases FLAG-HTA9ub band escaped WB detection with anti-HTA9 antibody, which we think is a strong evidence for this modification taking place. It is true that these results suggest that K129 is the preferred target lysine, and that we cannot rule out that

K132 is also monoubiquitinated to some extent. We have now clearly stated this in the revised version (see page 4 of new version). In any case, we think that the fact that K129, K132 or both could be monoubiquitinated would not change the main conclusions of the work. We hope Reviewer will agree.

Regarding the question: are H2Bub and HTA9ub really the only ubiquitinated species in this histone-enriched extract? Where is H2Aub? The anti-ubiquitin antibody is able to recognize HTA9ub, H2AK121ub and H2Bub in WT histone extracts; however, it is true that this was difficult to appreciate in the WB that we presented as the amount of WT sample loaded was not enough to show HTA9ub and H2AK121ub discrete bands. We apologize for this. We have now included a new WB in which this can be easily appreciated (See new Fig. 2c).

2. With respect to complementation of hta9/hta11 mutants with the FLAG-HTA9_N construct, the data suggest that this is not a complete rescue, which confounds some of the subsequent interpretations. For example, the early flowering of hta9/hta11 is known to result from lack of expression of FLC in the absence of H2A.Z deposition. Thus, the fact that hta9/hta11/ FLAG-HTA9_N does not completely restore normal flowering time (Fig 1D and E), suggests that the fusion protein is not fully functional. Consistent with this notion is the fact that many genes that require H2A.Z for activation are not properly expressed in the hta9/hta11/ FLAG-HTA9_N plants (Supplemental Figure 4B). If N-terminal acetylation is important for activation, then this lack of complete complementation may be explained by having an epitope tag at the N-terminus. The authors should at least consider this in their interpretations of the data.

We thank Reviewer for making this comment. We have revised this part of the manuscript according to Reviewer suggestions (see page 5 of the new version). In addition, we have included a figure (Fig.1g) showing *FLC* expression levels in the different plants and contemplate the possibility that the tag at the N-terminus might partially interfere with the proper functioning of H2A.Z in transcriptional activation.

3. Related to Figure 2E, and Page 5 paragraph 2, it is not reasonable to conclude anything about H2AK121ub being able to functionally replace H2A.Zub based on these experiments. We don't know that these histones were chromatin bound or if H2AK121ub and H2A.Zub are even at the same sites. This part of the text needs modification.

According to Reviewer suggestions, we have revised this part of the text. We have included in new Fig. 3d a Venn diagram showing the number of overlapping genes when comparing upregulated genes in *hta9hta11*, H2A.Z enriched genes in WT and H2AK121ub marked genes in WT. The result showed that both H2A.Z and H2AK121ub are present at the majority of the genes that became upregulated in *hta9hta11* in WT seedlings. For this reason we analyzed if the levels of H2AK121ub were altered in *hta9hta11*. We found increased levels in *hta9hta11* mutant. Since both H2A.Zub and H2AK121ub incorporation requires AtBMI1 activity, it might be possible that in absence of H2A.Z AtBMI1 activity tries to compensate for the loss of H2A.Zub by increasing

H2A monoubiquitination, which is in agreement with the increased levels of H2AK121ub in *hta9hta11*; however, the fact that *hta9hta11* mutants display misregulation of a considerable number of genes suggests that the increased levels of H2AK121ub cannot replace the role of H2A.Zub in regulating these genes. With this, we wanted to highlight that H2AK121ub and H2A.Zub play independent roles.

4. Based on Figure 4, the authors conclude that H2A.Z and H3K27me3 are essentially independent of one another at a functional level. However, the authors do not discuss recent results that seem to show interdependence between H2A.Z deposition and H3K27me3 deposition (Carter et al [2018] Plant Cell 30). The apparent contradiction between those results and the present manuscript must be discussed.

This is another important point. According to Reviewer suggestion, we have included the following discussion in the manuscript:

In contrast to our results, a recent report[33] showed that the levels of H3K27me3 were altered in *pie1* mutant, in which the incorporation of H2A.Z is impaired[12,33]. This finding led to propose that PIE1 acts with CURLY LEAF (CLF), which is one of the PRC2 H3K27 trimethyltransferases[34], in promoting H3K27me3 at a common set of genes. However, *pie1* mutants do not fully phenocopy seedlings severely depleted in H2A.Z[2,5], indicating that PIE1 have additional roles beyond H2A.Z deposition[2,5] which might affect H3K27me3 levels. The same report showed that *clf* mutant displayed strongly reduced H2A.Z levels, which was proposed to be a result of an H3K27me3-independent mechanism. Thus, it might be possible that the decreased levels of H3K27me3 at upregulated genes in *pie1* mutants are also an indirect effect of the activation of these genes. In agreement with this, another report[35] showed that although the transcriptional activation of anthocyanin biosynthesis genes is associated with reduced levels of H2A.Z and H3K27me3, altered levels of H3K27me3 are not associated with changes in the expression of these genes, supporting that the role of H2A.Z in repression is independent of PRC2 activity.

5. Regarding Figure 5A, it is very hard to believe that the S. pombe H2A.Zac antibody reacts with H2A.Z from Arabidopsis given the differences in amino acid sequence. Based on the weak bands shown on this western blot, I am not convinced. Further, if it is in fact reacting with acetylated H2A.Z, is it mono-, di-, tri-, or tetracetylated? There are ways to test this more directly, but I am not sure it is worthwhile in the context of this manuscript.

According to Reviewer comment, we agree that the part of H2A.Z acetylation is undeveloped and out of the scope of the manuscript. Since extensive additional data would be need for a proper analysis of H2A.Z acetylation, we have removed this part from the manuscript.

In any case, although western blot analysis using anti-H2A.Zub Ab from *S. pombe* showed much higher signal of H4ac than of H2A.Zac, the antibody

seems to recognize both acetylated histones in Arabidopsis. The antibody has been raised against an N-terminal peptide from *S. pombe* H2A.Z acetylated at K5, 7, 12 and 16. Despite the sequence differences between H2A.Z in Arabidopsis and *S. pombe*, the amino acids surrounding the acetylated lysines (GKG) in *S. pombe* are also found in Arabidopsis H2A.Z (HTA8, HTA9 and HTA11) and especially in H4 but are not found in H3, which may be the epitope recognized by the antibody.

6. Finally, on page 8 the authors conclude that modifications of H2A.Z at the +1 nucleosome are all that matters with respect to transcriptional regulation. I do not see any data that directly support this claim.

We have now included new data supporting that H2A.Zub may be located at +1 nucleosome of target genes and that this modification is decisive for H2A.Z-medited transcriptional repression (page 7-8 and page 11-12 of the new version, respectively). We found that H2A.Z signal at +1 nucleosome region represented 16.8% of total H2A.Z signal at the genes that become upregulated in *hta9hta11* (Supplementary Dataset 3), which fitted with the percentage of HTA9ub calculated by WB (Supplementary Fig. 1b). In addition, We show that the incorporation of either FLAG-HTA9_N or FLAG-HTA9_RR compensates for the low levels of HTA9 in *hta9hta11* mutants (Fig. 6; Supplementary Fig. 6), however, the repression of the genes upregulated in *hta9hta11* only occurs when FLAG-HTA9_N is incorporated (page 12; Fig. 6, Supplementary Fig. 6), indicating that is H2A.Z monoubiquitination, more than H2A.Z incorporation, what is decisive for H2A.Z-mediated repression. We hope that these new data support this claim.

Response to Reviewer #2

1. The authors claim that „ the early flowering phenotype of hta9hta11 was rescued in hta9hta11/FLAG-HTA9_N plants but not in mutants expressing FLAG-HTA9_RK or FLAG-HTA9_RR (Fig. 1d,e), which was supported by FT transcript levels (Fig. 1f)." To make the claim that the phenotype of hta9hta11 was rescued in hta9hta11/FLAG-HTA9_N plants, they should compare the difference between wild-type and hta9hta11/FLAG-HTA9_N; which based on the data will be significant; they can therefore only conclude that there was a partial rescue. In addition, the second claim is not correct, since there is a significant difference between hta9hta11 and FLAG-HTA9_RK or FLAG-HTA9_RR expressing plants; they can therefore only conclude that both constructs do not fully complement the phenotype of the mutant. Furthermore, the FLAG-HTA9_RR line clearly differs from the hta9/11 mutant, as evident by the data shown in Figure 3.

We thank Reviewer for making this comment. We have revised this part of the manuscript according to Reviewer suggestions and discussed differences among the different genotypes (see page 5 of the new version). In addition, we

have included a figure (Fig.1g) showing *FLC* expression levels in the different plants and contemplate the possibility that the tag at the N-terminus might interfere, at least partially, with the proper functioning of H2A.Z in transcriptional activation. As Reviewer indicates, *hta9hta11/FLAG-HTA9_RR* clearly differs from the *hta9/hta11* mutant since FLAG-HTA9_RR can be incorporated into chromatin and at least partially complements the role of H2A.Z in transcriptional activation.

2. Figure 3a: The comparison is highly confusing. Why did the authors not directly compare FLAG-HTA9_N with FLAG-HTA9RR? They should also reverse the order of the comparison and show hta9hta11 versus FLAG-HTA9_N; so that is clear that upregulated genes are upregulated as a consequence of impaired H2A.Z function.

We agree with Reviewer that it can be a bit confusing. We have performed the comparisons in this order because we wanted to show the genes that change their expression levels in *hta9hta11/FLAG-HTA9_N* or *_RR* compared to *hta9hta11*. Since all plants have the same background and only differ in the presence or absence of FLAG-HTA9_N or _RR transgene, we consider that the comparisons in this order provide a general view of effect of *FLAG-HTA9_N* or *_RR* expression in an *hta9hta11* background. The genes showed in red are the ones that become downregulated and in green upregulated in *hta9hta11/FLAG-HTA9_N* or *_RR* compared to *hta9hta11*. However, as suggested by the Reviewer, we have included the comparison of *hta9hta11/FLAG-HTA9_N* with *_RR* in Supplementary Fig. 4a, which shows that the transcriptome of *hta9hta11/FLAG-HTA9_N* and *hta9hta11/FLAG-HTA9_RR* mostly differ in the number of downregulated genes. We have now tried to explain more clearly the figure in the text. We hope that the changes will facilitate its understanding.

3. A study on mouse ES cells showed that ca. 25% of H2A.Zub is also acetylated (Ku et al. 2012). Another study showed that the difference in acetylation versus non-acetylation explains the difference in effect on transcription (Vandés-Mora 2012). The authors could also use (reciprocal) IP followed by WB to determine whether this double-marking is the case in Arabidopsis. If these modifications turn out to co-occur in the majority of the cases, then the model is not as clear-cut as they suggest.

We have removed the part regarding H2A.Zac from the manuscript as we agree with several comments coming from Reviewers indicating that this part is undeveloped and a bit out of the scope of the manuscript. In any case, to perform the experiments suggested by the Reviewer it would be required specific anti-HTA9ub and anti-HTA9ac antibodies, which unfortunately are not available at this moment. Nevertheless, we could speculate that double-marking at H2A.Z would not change the main conclusions of the work as the presence of H2A.Zub could repress gene expression even if H2A.Zac marks were present.

• *Page 3, top. Is the indication that H2A.Zub is about 20% of H2A.Z based on quantification?*

Yes, we have now included a bar chart showing the quantification in Supplementary Fig. 1.

• *The authors have an anti-H3 IP (protein immunoprecipitation) method in their methods section, but I cannot see any result obtained with such IP.*

The result was showed in Supplementary Fig. 2b. To verify that the FLAG-tagged proteins were incorporated into chromatin, we performed a WB using anti-HTA9 antibody on chromatin immunoprecipitated with anti-H3 antibody.

• *Subjective wording/rephrasing:*
-"very similar phenotype" on page 2. Remove the word "very".

Corrected

• *-"small fraction", change to minor fraction (as opposed to major)*

Corrected

• *-Figure1f, multiplication signs lacking on Y-axis labels.*

Corrected

• *-Figure 4b requires more intuitive labeling*

We have now clarified in the text and Figure legend that genes enriched in H2A.Z and marked with H3K27me3 in WT are labeled as H2A.Z/H3K27me3_WT. We hope that the changes will facilitate the understanding of Figure (now Fig. 5b) labeling.

• *-In Figure 4c: are the 3 upregulated and 3 downregulated genes part of the overlapping fields in Figure 4b? This is not immediately clear from the text.*

Yes, we selected three upregulated and three downregulated genes among the overlapping genes. This is now clarified in the text.

• *-Page 4: throughout plant developmental*

Corrected

• *-Page 4: PRC1 acts together PRC2*

Corrected

Response to Reviewer #3

The authors identified monoubiquitination of K129 of H2A.Z as a modification important for this histone variant, which can be partially responsible for repressive impact on the transcription. Furthermore, they identified the major enzyme responsible for the modification. The data are convincing and I have no objections to the presentation of this part of the manuscript.
On the other hand, the part about H2A.Z acetylation is undeveloped. The western analysis using anti-AC Ab shows much higher signal from H4 than from H2A.Z. I understand the authors explanation, but this result is not suitable for publishing – different, more specific Ab should be

generated. This part is a bit out of scope of the paper. Since extensive additional data would need to be added for a proper analysis of histone AC, I would suggest to completely remove this part from the manuscript.

According to Reviewer comment, we agree that H2A.Z acetylation part is undeveloped. We have removed this part in the new version.

The authors suggest that H2A.Zub occurs at +1 nucleosome, however there is no clear evidence that this is the case. I would be happy to see some ChIP data showing that indeed ubiquitination occurs predominantly at +1 nucleosomes.

This is an important point. As Reviewer indicates, there is no direct evidence for H2A.Zub occurring at +1 nucleosome. Unfortunately, an antibody that specifically recognizes HTA9ub is not available, thus, it is not possible to have direct evidence of its localization at this moment. However, we have tried to predict the possible localization of H2A.Zub by comparing H2A.Z signal in +1 nucleosome region with total H2A.Z signal (entire gene) in WT seedlings at the genes that become upregulated in *hta9hta11* (see page 7-8 of the new version; Supplementary Fig. 3b). This was estimated by summing the area under the curve (AUC) of each gene from TSS to 200 bp downstream TSS for +1 nucleosome region, and from TSS to TES for the entire gene (Supplementary Dataset 3). We found that H2A.Z signal at +1 nucleosome region represented 16.8% of total H2A.Z signal, which fitted with the percentage of HTA9ub calculated by WB. This is consistent with a model assuming that H2A.Zub is located at +1 nucleosome of repressed genes. However, we clearly indicated that further experiments will be required to rule out a different localization or even a spread distribution along the whole gene length.

The authors generated a really nice tool to study H2A.Z ubiquitination, FLAG-HTA9_RR and FLAG-HTA9_N lines, which is however not fully exploited in the context of the atbmi1 triple mutant. Performing some ChIP analysis of HTA9 for both constructs in the background of the atbmi1 triple mutant would provide information whether H2A.Z ubiquitination is crucial for the removal of H2A.Z from the nucleosomes upon transcriptional activation. I understand, however, that this experiment would require generating multiple mutant lines (hta9/11 atbmi1a/b/c) which needs to carry HTA9 transgenes. Therefore, I leave the decision whether this experiment is required to the competence of the Editors.

Thanks to Reviewer suggestion, we designed new experiments to determine if is H2A.Z monoubiquitination, more than H2A.Z incorporation, what is decisive for H2A.Z-mediated repression, and if the different levels of H2A.Z along activated and repressed genes may be a consequence of gene activity rather than an active mechanism to establish repression (see page 11-12 and Fig. 6). We investigated the levels of HTA9 in *atbmi1a/b/c* at genes that become downregulated and upregulated in the mutant and found increased and decreased levels of HTA9, respectively (Fig. 6b). This might be in agreement with a repressive role of H2A.Z incorporation in transcription. However, we

found similar levels of HTA9 in WT, *hta9hta11/FLAG-HTA9_N* and *hta9hta11/FLAG-HTA9_RR* plants at *HSP70* and *FLC* in which the incorporation of H2A.Z has been proposed to have a repressive and a promoting effect on transcription, respectively (Fig. 6c), indicating that the incorporation of either FLAG-HTA9_N or FLAG-HTA9_RR properly compensates for the low levels of HTA9 in *hta9hta11* mutants (Supplementary Fig. 6c); Nevertheless, while *FLC* expression was activated in both lines (Fig. 1g), a decrease in *HSP70* expression was only observed when *FLAG-HTA9_N* was incorporated (Supplementary Fig. 4), supporting that the incorporation of H2A.Z by itself is not an active mechanism to establish repression. The repressive effect of H2A.Z is only observed when a HTA9 version susceptible for monoubiquitination is incorporated.

Some conclusions in the ms are overstated and do not find confirmation in the data (please see below for details). The model proposed by the authors is interesting however too speculative. First, placing H2A.Zub in the +1 nucleosome is not supported in the data. Second, the role of H2A.Z acetylation in activating gene transcription is not supported at all. I suggest removing the right-hand part of the model and change H2A.Z ubiquitination based on new data, which should be provided. It would be good to improve images of nucleosomes (especially the DNA wrapping is very weird).

To make our model less speculative, we have included new data supporting that H2A.Zub may be located at +1 nucleosome region (see page 7-8 and Supplementary Dataset 3). We have also removed the H2A.Zac part from the model but we have left the right part to illustrate that actively transcribed genes display reduced levels of H2A.Z, which is supported by metagene plot and ChIP-qPCR results. In addition, we have improved the image of DNA and nucleosomes.

In general, this is an important study as it provides some clues about how H2A.Z histone variant can adopt different, antagonistic roles in regulation of transcription. From this perspective I believe it would be of interest for Nat Comm. readers and I recommend accepting the ms after revision.

Specific comments:

Fig. S2A. How the authors would explain the fact that WT HTA9 show at least 3x higher level of its modified version in FLAG-HTA9-N line that in FLAG-HTA9-RK and -RR lines? I am afraid whether the lack of the modified version of FLAG-HTA9 in those two lines could be due to apparent decreased detection level in the two mutated lines.

As the reviewer indicates, the higher levels of endogenous modified HTA9 in *hta9hta11/FLAG-HTA9-N* than in *hta9hta11/FLAG-HTA9-RK* and -RR lines are due to differences in loading amounts. We apologize for not including the WB probed with anti-H4 for loading control (we have now included it). In any case,

Fig. 1c clearly shows the complete absence of modified FLAG-HTA9-RK and –RR band in WT background when comparing similar amounts of the different histone extracts. Therefore, despite differences in loading amounts in Supplementary Fig. 2a, there is no reason to expect that the modification of FLAG-HTA9-RK or –RR take places in an *hta9hta11* background.

p. 4 "We found that the early flowering phenotype of hta9hta11 was rescued in hta9hta11/FLAG-HTA9_N plants but not in mutants expressing FLAG-HTA9_RK or FLAG-HTA9_RR (Fig. 1d, e), which was supported by FT transcript levels (Fig. 1f)." According to the results shown in Figs. 1d, e, f the phenotype of FLAG-HTA9_RK and _RR lines also seems to be improved. The authors should indicate this, even though it is not statistically significant, as the data for both types of experiments (flowering time and FT expression) and both lines are very consistent.

We thank Reviewer for making this comment. We have revised this part of the manuscript accordingly (see page 5 of the new version). In addition, we have included a figure (Fig.1g) showing *FLC* expression levels in the different plants.

p. 4"This activity is required to maintain gene repression throughout plant developmental." Should be "plant development"

Corrected

p.4 "atbmi1a/b/c mutants remain in an embryo maturating-like phase due to misexpression of the embryonic program after germination25 (Supplementary Fig. 3). Moreover, 20% of Arabidopsis transcriptome is misregulated in these mutants" This is one triple mutant or several different triple mutants? In the first case, change the plural for singular. Please, change accordingly in the whole ms.

It is one triple mutant. We have changed accordingly throughout the manuscript.

p.4 "In fact, PRC2 to incorporate H3K27me3 requires the presence of H2AK121ub marks at these genes31" change the word order to "In fact, PRC2 requires the presence of H2AK121ub marks to incorporate H3K27me3 at these genes31"

Corrected

p. 5"Since HTA9ub represented a small percentage of total HTA9, it was reasonable to assume that it localizes at +1 nucleosome region." This is a wide speculation, in addition based on wrong assumptions. It is well-known that H2A.Z localizes predominantly at +1 nucleosome; this location constitutes about 80% of total H2A.Z or even more. Therefore, the authors should argue that it resides in gene body rather than in +1, if they want to use the argument of similar percentage, which in general I think is irrational.

As Reviewer indicates, H2A.Z localizes more frequently at +1 nucleosome region. This is reflected as a peak at this region in metagene plots (Fig. 3b and c); however, the overall enrichment of H2A.Z at gene bodies is also high, particularly at repressed genes. This indicates that a considerable number of

nucleosomes along gene bodies contains H2A.Z although with a random distribution. Since HTA9ub represented around 20% of total HTA9 (see new Supplementary Fig. 1b), we hypothesized that H2A.Zub marks may have a local distribution as a widespread distribution along genes would represent a higher percentage of total HTA9. Therefore, a reasonable possibility could be that H2A.Zub localizes at +1 nucleosome region. To evaluate this, we estimated H2A.Z signal in +1 nucleosome region and compared it to total H2A.Z signal (entire gene) in WT at the genes that become upregulated in *hta9hta11* (see page 7-8). We found that H2A.Z signal at +1 nucleosome region represented 16.8% of the total H2A.Z signal (Supplementary Dataset 3), which fitted well with the percentage of HTA9ub calculated by WB. This is consistent with a model assuming that H2A.Zub is located at +1 nucleosome of repressed genes. However, further experiments will be required to rule out a different localization or even possible spread distribution of H2A.Zub marks along the whole gene length. We have included these new results and discussion in the new version of the manuscript.

p.5 and Fig. 3c and d "Venn diagrams showing the percentage of genes upregulated in hta9hta11 that are not upregulated in hta9hta11/FLAG-HTA9_N (left panel)" I understand that the diagrams show up- and down-regulation of genes in the three lines when compared to wt? If this is the case, it should be clearly stated in the figure caption.

Yes, the different subsets of genes correspond to up- and downregulated genes when compared to WT. We have now clarified this in the text and figure legend (now Fig. 4).

In addition:
Fig. S4. The way of data presentation suggests RT-qPCR? Only after checking the units on the axis you can find that this is RNA-seq data. This should be made more clear in the figure caption.

We have now increased the size of y-axis labeling to clearly show the units and indicated that those are RNA-seq data in the Figure legend.

p. 8"(…) since H2A.Z levels at repressed genes are higher than at active genes in WT (Fig. 2f), it might be possible that the increased levels of HTA9 in atbmi1a/b/c are a consequence of the high number of downregulated genes." I do not understand this conclusion, since the number of upregulated genes in atbmi1 triple is higher than downregulated ones. I would rather speculate that increased amount of HTA9 in the mutant is a way to compensate the lack of ubiquitination since both the H2A.Zub and H2A.Z alone show repressive impact on transcription.

WB results showed that HTA9 levels were significantly increased in *atbmi1a/b/c* compared to WT, even considering the monoubiquitinated fraction included (Fig. 2a,d,3e). *atbmi1a/b* weak and *atbmi1a/b/c* mutants displayed a very high

number of upregulated genes (Fig. 6a; Supplementary Dataset 2 and Supplementary Dataset 5), which is consistent with a loss of PRC1 repression; however, unlike *atbmi1a/b* weak, *atbmi1a/b/c* mutant showed a higher number of downregulated genes than upregulated (Fig. 6a; Supplementary Dataset 2 and Supplementary Dataset 5). We proposed that the high number of downregulated genes in *atbmi1a/b/c* might explain the globally increased levels of HTA9 in mutant compared to WT as we found that the levels of HTA9 were increased at repressed genes in *atbmi1a/b/c* (Fig. 6b, left). As Reviewer indicates, the incorporation of H2A.Z along genes has been proposed to have a repressive role in transcription[7,8], which might be in agreement with the high levels of HTA9 found at repressed genes in *atbmi1a/b/c*. However, our new results indicated that the incorporation H2A.Z by itself does not seem to have a repressive role (see page 12; Fig. 6 and Supplementary Fig. 6) as gene repression was only achieved when a *FLAG-HTA9* susceptible for monoubiquitination *(FLAG-HTA9_N)* was incorporated into an *hta9hta11* background. This, together with the fact that the levels of H2A.Z at repressed genes are higher than at active genes (see Fig. 3b and c), led us to propose that the levels of H2A.Z along genes may be a consequence of gene activity rather than an active mechanism to establish repression. Interestingly, this has been also recently proposed in animals (Lashgari et al., 2017).

p.8"On the other hand, the levels of H2A.Z at genes are a consequence of the transcriptional activity." I think that this is overstated. I don't see any evidence in the authors' data that changes of H2A.Z level during transcriptional activation and repression are consequences of the transcription (although I think this is likely).

As we mentioned above, our new results indicate that the incorporation H2A.Z by itself does not seem to have a repressive role (see page 12; Fig. 6 and Supplementary Fig. 6). This, together with data showing that gene repression leads to increased H2A.Z levels while gene activation to decreased (Fig. 3 and Fig.6), suggests that the different levels of H2A.Z along genes may be a consequence of gene activity rather than an active mechanism to establish repression. We hope that the new evidence that we present will address Reviewer concerns in this respect.

Reviewers' comments:

Reviewer #1 (Remarks to the Author):

The revised manuscript by Gomez-Zambrano is much improved in many ways, but I am not entirely satisfied with their responses to several of the points I raised.

1. In my original review I noted that the evidence for ubiquitination of HTA9, while quite strong, remains circumstantial rather than direct. It is hard to imagine another modification to HTA9 causing it to run so slowly in SDS-PAGE, but nevertheless it would have been ideal to demonstrate ubiquitination directly (regardless of which specific residue it might be on). My suggestion was simply to perform an IP for HTA9 followed by a western blot for Ubiquitin, or some variation thereof. This is not absolutely critical, but I would certainly give it a try if this were my manuscript.

2. Another point I raised was the apparent contradiction of the presented findings with those from Carter et al. [2018] Plant Cell 30, who found that disruption of H2A.Z deposition led to loss of H3K27me3 deposition, and vice versa. Gomez-Zambrano et al argue that depletion of H3K27me3 in pie1 mutants could be attributable to increased transcription of those genes in the mutant, and this is plausible. But how can we explain the loss of H2A.Z deposition in the clf mutant observed by Carter et al if there is no functional connection between H2A.Z and PRC2? In addition, other laboratories have reported such a connection between H2A.Z and PRC2 in other organisms (Creyghton et al. [2008] Cell 135; Wang et al. [2018] BMC Biol 16.]. Perhaps there is an explanation for the apparent dissonance in these findings, but it remains unclear. The fact that the H2A.Z and H3K27me3 datasets used in this study are from different labs, slightly different growth conditions, and plant ages also raises some doubt. It is hard to imagine how H2A.Zub-mediated transcriptional repression is independent of PRC2 if deposition of H2A.Z is generally dependent on PRC2. At the very least a more thorough discussion of these issues is warranted.

3. As I pointed out in the original review, the authors conclude that modifications of H2A.Z at the +1 nucleosome are all that matters with respect to transcriptional regulation. The current revision posits that because ~20% of HTA9 is modified and ~20% of HTA9 is found around the +1 nucleosome at genes upregulated in hta9/hta11, that the +1 nucleosome must be the one that is ubiquitinated. I do not believe that this correlation supports that conclusion. In lieu of an HTA9Ub antibody, one approach to address this would be to perform sequential ChIP using the HTA9 antibody first and the Ubiquitin antibody second. Otherwise, it would be better to avoid this topic without further experimental data.

Overall, I find this manuscript highly intriguing and important, but I think there a few points that still need to be addressed.

Reviewer #2 (Remarks to the Author):

The manuscript has been improved, but a number of issues remain to be addressed. There are two main flaws; the first one is the claim about HTA9ub being at the +1 nucleosome. Despite the authors' arguments, I still do not see evidence supporting this claim. My suggestion is to remove this particular claim from the manuscript. The second flaw is the relationship between H2A.Z and expression. The authors claim that the HTA9ub (at +1 nucleosome position) is required for repression, while general (gene-body) incorporation of H2A.Z is a consequence of the transcription level (high transcription results in low H2A.Z). However, the authors have made some confusing analyses that do not test exactly the thing they want to test, and then subsequently the authors draw the wrong conclusions from these analyses. So far, they have shown this pattern in a couple of hand-picked genes, but to make general claims the authors ought to refine some of their analyses.
Specific comments:

1. The fact that the amount of HTA9 found at the +1 nucleosome is approx. 20% and the fact that the amount of modified (mono-ubiquitinated) HTA9 is also approx. 20% does not mean that the one equals the other. A second argument that the authors give to support this claim is that the modification is required for repression at a subset of genes. This also does not mean that the modification has to be at the +1 nucleosome, as repressors can occur elsewhere. As they have not provided any evidence of preferential HTA9ub localization, they should remove all mention of it.

2. Using data shown in Figure 4 und Suppl. Figure 4, the authors aims to address the questions (i) to what degree do genes that are upregulated and downregulated in the hta9hta11 mutant return to normal in the hta9hta11 FLAG-HTA9, and (ii) what are the differences in this rescue between FLAGHTA9-N and FLAG-HTA9-RR. The problem with figure 4a (and supplemental fig 4a) is that the genes that are upregulated in the rescue line compared to the mutant do not have to be the same as the genes that are downregulated in the mutant compared to wt. And even if they are the same (which is impossible to tell with this analysis), they may not return to wt level completely, or deregulate in the other direction. The problem with figure 4c and 4d is that the up- (or down-) regulated genes in the mutant that are not up-regulated (or down-) in the rescue line, are not necessarily returned to wt level. They can have become deregulated in the other direction.

One solution is to make a figure that shows the differential expression (hta9hta11 FLAG-HTA9 / wt) on the y-axis and make boxplots of genes defined by differential expression in hta9hta11 / wt (say downregulated, stable, and upregulated.). That way one can tell whether the genes upregulated in the mutant are also upregulated in the rescue line, or whether they have returned to wt level. Instead of using three categories one could also rank the genes based on expression (mutant /wt) and divide these into 10 equal groups, and make a boxplot for each group.

Another possible figure would be to make new Venn-diagrams, but instead of overlapping 'up' in mutant vs 'up' in rescue, they could compare 'up' in mutant versus 'stable' in rescue. That way they could really say what % returned to normal level, and properly compare the two constructs.

Minor points:
1. Line 148-152/ Figure 3a: To be able to use the word 'enriched' here, the authors need to statistically test whether the groups of genes upregulated are more likely to be marked with H2A.Z than other regions

2. Line 159: "very high" and "considerable" are imprecise terms. If the authors should include some kind of analysis that shows what percentage of nucleosomes possess H2A.Z.

3. Line 168-169: the percentage (20%) does not indicate thin vs wide distribution. Because always a mixture of cells is investigated, it could be that only 20% of cells have this mark on all nucleosomes containing H2A.Z along the gene body.

4. Line 201: "considerable" is imprecise, add % information.

5. Line 202: "substantial" is imprecise. Mention the percentages of genes going up/down or returning to wt level.

6. Lines 203-205: The text is unclear about what the change is. The authors write that the two lines (hta9hta11/FLAG-HTA9_N and hta9hta11/FLAG-HTA9_RR) differ in the number of downregulated genes and then refer to sup figure 4a. Firstly, if they want to mention this difference they ought to say

which one has more/less, and by how much. Second, the figure 4a does not fit the statement. The figure is a volcanoplot that shows that the -N line has more downregulated genes than upregulated genes, when comparing to the -RR line.

7. Line 205/209: "significant" and "considerable", change to minor/major or %.

8. Line 205-211 & figure 4c/d: Firstly, the figures are not interpreted correctly. The genes indicated with arrows don't have to be genes returning to wt expression level. For upregulated genes, for instance, it can be that the indicated genes are downregulated in the rescue line compared to wt. Secondly, the authors consider the 19% of genes upregulated in the mutant but not upregulated in the rescue line to be insignificant, but the authors do not provide a statistical argument. The only thing that the authors could say is that the -N line has a higher percentage of non-recovered upregulated genes than the -R line, and that the -RR line has a higher percentage of non-recovered downregulated genes than the -N line.

9. Line 262: "very high”, put precise %.

10. Line 277-282: This is not true, or at least, the figures provided (figure 4) do not show this. New figures need to be provided to re-examine the claims.

11. Line 291: no the results do not. The results about HTA9ub are only clear on the FLC and FT loci, but this is hardly enough to make sweeping claims. New analyses should be provided (as indicated above) to make proper claims about the function of HTA9ub in repression rather than activation.
12. Line 302: remove word "reduces"

13. Line 310/311: the authors have not shown that the level of H2A.Z in genebody is a consequence of transcription. Part of the problem is that the claim that HTA9ub is on +1 does not hold, and so this may occur along the genebody, explaining the negative correlation. Secondly, the authors may have indicated (and with some refined analyses in the future one may say shown) that HTA9ub is important for H2A.Z mediated gene repression, but they did not show it is 100%. The construct with the mutated K has still half the effectivity of the non-mutated one (though again, this is based on an incorrect analysis) 19% compared to 36%, indicating that some repressive ability still exists. As it stands the authors can only say that modified HTA9 has a larger role in repression than unmodified HTA9.

14. Figure 3b. It would be better to use a color gradient to indicate transcript amount. (like from light yellow for lowly expressed to dark red for highly expressed)

15. Figure 6c. Results of statistical tests missing.

16. Sup fig 2A could use some annotation of the expected identity of the different bands
17. Supplementary figure 4b. Results of statistical tests missing. And odd dots are present next to error bars that don't seem to have any purpose.

18. General: it would be good that the authors indicated which genes they included in their analysis. Whether they looked at all nuclear genes, or only protein-coding genes. And also how many genes were detected in the RNAseq experiments. Firstly it would be good to know in general on what kinds of genes the results are obtained from. But especially for the venn-diagrams it would be easier to

determine how likely it is to obtain overlaps by chance (and whether there are over or under enrichments.

Reviewer #3 (Remarks to the Author):

I must say I was a bit disappointed with the new version of the manuscript. The major change was removing the part of H2A.Z histone acetylation. The authors should know, however, that it is expected to add new data to the ms during revision, and not to remove them (though in this case this was suggested). Unfortunately, the authors added only one experiment (Fig. 6c), poor substitute for what was expected (see below). In my opinion, more work is required to warrant publication in Nature Communications.

I am not convinced at all with the way the authors tried to confirm +1 nucleosome location of H2A.Zub. I understand that @HTA9ub is not available, but it is possible to ChIP on BMI1 or at least H2AK121ub, not mentioning sequential ChIP on FLAG-HTA9 and then @Ub.

In the new Fig. 6c the authors compared the H2A.Z levels in two genes, FLC and HSP70, for which transcription H2A.Z has promoting and repressive effect, respectively. There are several problems with this experiment: First, there is no data that those genes are directly regulated by H2A.Z – one can imagine that reduced levels of H2A.Z in hta.z double mutant affect expression of some TFs, which in turn change FLC and HSP70 expression. Although FLC and HSP70 were extensively described in the context of H2A.Z, this was done 10 years ago or more, when our knowledge about H2A.Z role in transcriptional regulation was very limited, and direct effect was never proved for those genes. Therefore, the authors should include more than just one gene per each group, selecting genes from more recent works. Second, amplicons were not wisely chosen: the authors took amplicon +80 from Kumar & Wigge Cell 2010 paper instead of amplicon +119, which represents the center of +1 nucleosome, all other amplicons show even lower levels of H2A.Z, thus the signal may represent just a noise even when it is not observed in hta9 hta11 mutant shown in a supplementary file. In concordance with this, the second FLC amplicon selected by Gomez-Zambrano et al. lies in the middle of long intron and it is known to have no H2A.Z (see eg Deal et al. Plant Cell 2007), while the authors can still retrieve significant enrichment. The negative control from Suppl. Fig. 6e should be presented along with the other line, otherwise it is misleading. Why the authors did not selected amplicons based on available browsers showing H2A.Z distribution in a genome-wide manner? I think that this experiment should be extended for other genes presented in Fig. 6b, providing that their also differ in transcriptional behavior in hta9 hta11 (or at least in arp6 mutant). Another option would be to add some genes extensively studied in the context of H2A.Z distribution, eg some genes presented in Sura et al. Plant Cell 2017 paper. This would provide an opportunity to extend their conclusions with the authors new data.

Response to Reviewers

Reviewer #1

The revised manuscript by Gomez-Zambrano is much improved in many ways, but I am not entirely satisfied with their responses to several of the points I raised.

1. In my original review I noted that the evidence for ubiquitination of HTA9, while quite strong, remains circumstantial rather than direct. It is hard to imagine another modification to HTA9 causing it to run so slowly in SDS-PAGE, but nevertheless it would have been ideal to demonstrate ubiquitination directly (regardless of which specific residue it might be on). My suggestion was simply to perform an IP for HTA9 followed by a western blot for Ubiquitin, or some variation thereof. This is not absolutely critical, but I would certainly give it a try if this were my manuscript.

According to Reviewer suggestion, we have immunoprecipitated HTA9 from WT chromatin and then performed a WB using anti-ubiquitin antibody to detect HTA9ub. We have had serious cross-reaction problems with the antibody used for IP, especially with the light chain of the ab as it has a similar MW than monoubiquitinated HTA9. We have tried the IP with FLAG and HTA9 antibodies but we got the same results. We have placed in Supplementary Figure 3b our best result. Apparently, the ubiquitin antibody detects a band with the MW of HTA9ub; however, we cannot be sure that this band is indeed HTA9ub, therefore we labeled as "possible HTA9ub". In any case, we have previously demonstrated that AtBMI1 proteins are E3 monoubiquitin ligases *in vitro* and *in vivo* (Bratzel et al., 2010), and here we show that HTA9ub band could not be detected in *atbmi1* mutants and that the ubiquitin antibody recognizes HTA9ub in WT histone extracts but not in *hta9hta11*, which we think strongly support that the slow-migrating band of HTA9 is a monoubiquitinated form.

2. Another point I raised was the apparent contradiction of the presented findings with those from Carter et al. [2018] Plant Cell 30, who found that disruption of H2A.Z deposition led to loss of H3K27me3 deposition, and vice versa. Gomez-Zambrano et al argue that depletion of H3K27me3 in pie1 mutants could be attributable to increased transcription of those genes in the mutant, and this is plausible. But how can we explain the loss of H2A.Z deposition in the clf mutant observed by Carter et al if there is no functional connection between H2A.Z and PRC2? In addition, other laboratories have reported such a connection between H2A.Z and PRC2 in other organisms (Creyghton et al. [2008] Cell 135; Wang et al. [2018] BMC Biol 16.]. Perhaps there is an explanation for the apparent dissonance in these findings, but it remains unclear. The fact that the H2A.Z and H3K27me3 datasets used in this study are from different labs, slightly different growth conditions, and plant ages also raises some doubt. It is hard to imagine how H2A.Zub-mediated transcriptional repression is independent of PRC2 if deposition of H2A.Z is generally dependent on PRC2. At the very least a more thorough discussion of these issues is warranted.

According to Reviewer suggestion, we have included a more exhaustive discussion at this respect (see discussion section, page 16-17). Although we did not find significant

changes in global H3K27me3 levels between WT and *hta9hta11* or *arp6-10* (result included in the new version in Suppl. Fig 5) or at selected genes, it is true that these results cannot rule out a possible role of H2A.Z in modulating H3K27me3 deposition at other loci. However, it might be also possible that altered levels of one histone mark impact the levels of other/s, especially when they co-localize and the transcriptional activity of the gene change. We contemplated all these possibilities in the new version (please, see discussion of the new version).

Regarding the specific comment “It is hard to imagine how H2A.Zub-mediated transcriptional repression is independent of PRC2 if deposition of H2A.Z is generally dependent on PRC2”, we would like to point out that Wang et al. (2018) showed that in mouse ESCs H3K27me3 enrichment correlates strongly with H2A.Z and proposed that H2A.Z promotes PRC2 activity through facilitating chromatin compaction. Creyghton et al. (2008) found that H2AZ and PcG protein occupancy is interdependent at promoters, although they also showed that H3K27me3 and H2AZ occupy different subsets of genes in lineage-committed cells; Surface et al., (2016) proposed that H2A.Z.1ub promotes the association of PRC2 and H3K27me3 with bivalent promoters in murine ESCs; and Carter et al., (2017) propose that H2A.Z in Arabidopsis modulates H3K27me3 deposition. Therefore, it seems that H2A.Z could play a role in H3K27me3 deposition more than the other way around. This could be in line with recent reports in animals and plants showing that the incorporation of canonical H2Aub is independent of H3K27me3 but H2Aub is required for H3K27me3 at some targets (Blackledge et al., 2014; Kalb et al., 2014; Cooper et al., 2014; Yang et al., 2013; Zhou, Romero-Campero et al., 2017); Nevertheless, in Arabidopsis there is also a considerable number of genes only H2AK121ub marked, indicating that PRC1 activity can regulate gene expression independently of PRC2 (Zhou, Romero-Campero et al., 2017). On the other hand, Carter et al., (2017) propose that CLF and/or H3K27me3 are unlikely to be directly required for deposition of H2A.Z. They showed that H2A.Z in *clf* was reduced at genes that were not even marked with H3K27me3, suggesting an indirect effect. All together these data strongly suggest that deposition of H2A.Z is not dependent on PRC2.

In any case, what we wanted to highlight (maybe we did not do it clearly in the previous version) is that the transcriptional repressive effect of H2A.Zub is not dependent on H3K27me3, as most of the upregulated genes in *hta9hta11* (64%) were not marked with H3K27me3 in WT. In addition, loss of PRC2 activity does not lead to the upregulation of the same subset of genes than the loss of H2A.Z. In agreement with this, a recent report (Cai et al., 2019) showed that although the transcriptional activation of anthocyanin biosynthesis genes in loss of H2A.Z mutants is associated with reduced levels of H2A.Z and H3K27me3, the reduced levels of H3K27me3 are not associated with changes in the expression of these genes, supporting the prevailing role of H2A.Zub in the transcriptional repression in absence of H3K27me3.

It is true that H2A.Z and H3K27me3 datasets used in this study are from different labs, slightly different growth conditions and plant ages (10 DAG and 7 DAG, respectively); however, our expression data and H3K27me3 localization data were obtained from seedlings of the same age and under the same culture conditions and, if we compared the genes marked with H3K27me3 in WT and the genes upregulated in *hta9hta11* and *atbmi1a/b* weak (without considering H2A.Z enrichment) 70% of the commonly

upregulated genes in the two mutants are not marked with H3K27me3, supporting that H3K27me3 is not necessary for H2A.Z mediated repression.

3. As I pointed out in the original review, the authors conclude that modifications of H2A.Z at the +1 nucleosome are all that matters with respect to transcriptional regulation. The current revision posits that because ~20% of HTA9 is modified and ~20% of HTA9 is found around the +1 nucleosome at genes upregulated in hta9/hta11, that the +1 nucleosome must be the one that is ubiquitinated. I do not believe that this correlation supports that conclusion. In lieu of an HTA9Ub antibody, one approach to address this would be to perform sequential ChIP using the HTA9 antibody first and the Ubiquitin antibody second. Otherwise, it would be better to avoid this topic without further experimental data.

As Reviewer indicates, the correlation between the amount of HTA9ub and the H2A.Z signal at +1 nucleosome does not represent direct evidence of the localization of H2A.Zub at +1 nucleosome region. Accordingly, the Reviewer suggested to perform a sequential-ChIP experiment as an alternative way to determine if HTA9ub modification is located at +1 nucleosome of repressed genes in absence of an anti-H2A.Zub specific antibody; otherwise, recommended to avoid this topic.

Performing a sequential-ChIP, first using anti-HTA9 or -FLAG antibody and then anti-ubiquitin, will not provide a reliable result as we have to work in a chromatin context, in which nucleosomes are constituted by different histones that could also carry this modification. Thus, we can IP chromatin containing HTA9 or FLAG-HTA9; however in the second ChIP we will not be able to distinguish if the immunoprecipitated material results from the binding of the antibody to an ubiquitin residue present in HTA9 or in other histone. We have previously showed that canonical H2A can be monoubiquitinated by AtBMI1 to mediate gene repression (Bratzel et al., 2010; Yang et al., 2013; Zhou, Romero-Campero et al., 2017). Here we showed that H2A.Z enriched genes overlapped with H2AK121ub marked genes (11,804 genes out of 22,523 H2A.Z enriched genes were H2AK121ub marked; Fig. 3d); furthermore, most of the genes upregulated in *hta9hta11* were marked with H2AK121ub, indicating H2A.Z and H2AK121ub marks co-occupy a considerable number of genes. Since 80% of the H2AK121ub peaks overlap with the region immediately downstream the TSS of target genes (Zhou, Romero-Campero et al., 2017), it will be not possible to determine if the ubiquitin antibody is recognizing the mark incorporated into H2A.Z or into canonical H2A. Moreover, H2B can also be monoubiquitinated (Cao et al., 2008; Gu et al., 2009; Xu et al., 2009; Roudier et al., 2011). According to this, WB using anti-ubiquitin antibody on Arabidopsis histone extracts detected H2Bub, H2AK121ub and H2A.Zub (Fig. 2c). Sequeira-Mendes et al., 2014 defined several chromatin states in Arabidopsis based on the modifications and histone variants predominantly found in them. State 1 is characterized by high amounts of H3K4me3, H3 acetylation, H3K36me3, H2Bub and by nucleosome enriched in H3.3 and H2A.Z, which was typically associated with transcribed regions and TSSs. H2Bub and H2A.Z are also found in chromatin state 3, which is associated with transcriptionally elongating regions. Accordingly, H2Bub and H2A.Z may co-localize at different regions and thus again it will be not possible to determine if the ubiquitin antibody is recognizing the mark incorporated into H2A.Z or H2B.

Unfortunately, a reliable localization of H2A.Zub marks definitely requires a specific antibody that is not yet available. Therefore, following Reviewer suggestion, we have removed this part from the new version of the manuscript and only discuss this possibility in the discussion section (see page 15-16).

Overall, I find this manuscript highly intriguing and important, but I think there a few points that still need to be addressed.

Reviewer #2

The manuscript has been improved, but a number of issues remain to be addressed. There are two main flaws; the first one is the claim about HTA9ub being at the +1 nucleosome. Despite the authors' arguments, I still do not see evidence supporting this claim. My suggestion is to remove this particular claim from the manuscript. The second flaw is the relationship between H2A.Z and expression. The authors claim that the HTA9ub (at +1 nucleosome position) is required for repression, while general (gene-body) incorporation of H2A.Z is a consequence of the transcription level (high transcription results in low H2A.Z). However, the authors have made some confusing analyses that do not test exactly the thing they want to test, and then subsequently the authors draw the wrong conclusions from these analyses. So far, they have shown this pattern in a couple of hand-picked genes, but to make general claims the authors ought to refine some of their analyses.

Specific comments:

1. The fact that the amount of HTA9 found at the +1 nucleosome is approx. 20% and the fact that the amount of modified (mono-ubiquitinated) HTA9 is also approx. 20% does not mean that the one equals the other. A second argument that the authors give to support this claim is that the modification is required for repression at a subset of genes. This also does not mean that the modification has to be at the +1 nucleosome, as repressors can occur elsewhere. As they have not provided any evidence of preferential HTA9ub localization, they should remove all mention of it.

As Reviewer indicates, the correlation between the amount of HTA9ub and the H2A.Z signal at +1 nucleosome does not represent direct evidence of the localization of H2A.Zub at +1 nucleosome region. Unfortunately, a reliable localization of H2A.Zub marks definitely requires a specific antibody that is not yet available. Therefore, following Reviewer suggestion, we have removed this part from the new version of the manuscript and only discuss its possible localization in the discussion section (see page 15-16).

2. Using data shown in Figure 4 und Suppl. Figure 4, the authors aims to address the questions (i) to what degree do genes that are upregulated and downregulated in the hta9hta11 mutant return to normal in the hta9hta11 FLAG-HTA9, and (ii) what are the differences in this rescue between FLAGHTA9-N and FLAG-HTA9-RR. The problem with figure 4a (and supplemental fig 4a) is that the genes that are upregulated in the rescue line compared to the mutant do not have to be the same as the genes that are downregulated in the mutant compared to wt. And even if they are the same (which is impossible to tell with this analysis), they may not return to

wt level completely, or deregulate in the other direction. The problem with figure 4c and 4d is that the up- (or down-) regulated genes in the mutant that are not up-regulated (or down-) in the rescue line, are not necessarily returned to wt level. They can have become deregulated in the other direction.

One solution is to make a figure that shows the differential expression (hta9hta11 FLAG-HTA9 / wt) on the y-axis and make boxplots of genes defined by differential expression in hta9hta11 / wt (say downregulated, stable, and upregulated.). That way one can tell whether the genes upregulated in the mutant are also upregulated in the rescue line, or whether they have returned to wt level. Instead of using three categories one could also rank the genes based on expression (mutant /wt) and divide these into 10 equal groups, and make a boxplot for each group.

Another possible figure would be to make new Venn-diagrams, but instead of overlapping 'up' in mutant vs 'up' in rescue, they could compare 'up' in mutant versus 'stable' in rescue. That way they could really say what % returned to normal level, and properly compare the two constructs.

We thought that the Heatmap representation of expression levels of the misregulated genes in each genotype (Fig. 4d) was giving an idea to what degree the genes that were up and downregulated in the *hta9hta11* mutant return to normal in the *hta9hta11/FLAG-HTA9* lines. However, we would like to thank Reviewer for his/her suggestions as Fig. 4 has substantially improved and, importantly, reinforced our claims. We now show the number of genes misregulated in *hta9hta11* that recover WT levels in the two lines, and compared the differential expression in *hta9hta11* and in the *hta9hta11/FLAG-HTA9* lines displayed by the genes that, according our cutoff criterial (log2-fold change cut-off>|1|, pvalue <0.05), are commonly misregulated in mutant and _N or _RR lines. These results are shown in new Fig. 4, Supplementary Figure 4, and described in the new version of the manuscript (see results section, page 8-10).

Minor points:

1. Line 148-152/ Figure 3a: To be able to use the word 'enriched' here, the authors need to statistically test whether the groups of genes upregulated are more likely to be marked with H2A.Z than other regions

We have corrected this sentence specifying that "most of the upregulated genes in *hta9hta11* and *atbmi1a/b* weak mutants overlapped with the group of genes enriched in H2A.Z in WT".

2. Line 159: "very high" and "considerable" are imprecise terms. If the authors should include some kind of analysis that shows what percentage of nucleosomes possess H2A.Z.

Since we have removed the part in which we showed correlation between the amount of HTA9ub and the H2A.Z signal at +1 nucleosome, we have modified the complete paragraph (new lines 158-164).

3. Line 168-169: the percentage (20%) does not indicate thin vs wide distribution. Because always a mixture of cells is investigated, it could be that only 20% of cells have this mark on all nucleosomes containing H2A.Z along the gene body.

As we mentioned, we agree that the correlation does not represent direct evidence supporting the localization of H2A.Zub at +1 nucleosome region; therefore, following reviewer suggestion, we have removed this part from the manuscript. We will try to address this question in the future, as soon as appropriate tools are available (e.g. an anti-HTA9ub antibody).

4. Line 201: "considerable" is imprecise, add % information.

Corrected

5. Line 202: "substantial" is imprecise. Mention the percentages of genes going up/down or returning to wt level.

Corrected

6. Lines 203-205: The text is unclear about what the change is. The authors write that the two lines (hta9hta11/FLAG-HTA9_N and hta9hta11/FLAG-HTA9_RR) differ in the number of downregulated genes and then refer to sup figure 4a. Firstly, if they want to mention this difference they ought to say which one has more/less, and by how much. Second, the figure 4a does not fit the statement. The figure is a volcanoplot that shows that the -N line has more downregulated genes than upregulated genes, when comparing to the -RR line.

Since we have added more informative figures regarding transcriptome analyses, we have removed Supplementary Fig. 4a.

7. Line 205/209: "significant" and "considerable", change to minor/major or %.

Corrected

8. Line 205-211 & figure 4c/d: Firstly, the figures are not interpreted correctly. The genes indicated with arrows don't have to be genes returning to wt expression level. For upregulated genes, for instance, it can be that the indicated genes are downregulated in the rescue line compared to wt. Secondly, the authors consider the 19% of genes upregulated in the mutant but not upregulated in the rescue line to be insignificant, but the authors do not provide a statistical argument. The only thing that the authors could say is that the -N line has a higher percentage of non-recovered upregulated genes than the -R line, and that the -RR line has a higher percentage of non-recovered downregulated genes than the -N line.

We have now changed the figure to show the percentage of genes misregulated (up and down) in *hta9hta11* that recovered WT expression levels in *hta9hta11/FLAG-HTA9_N* and *hta9hta11/FLAG-HTA9_RR* lines (Fig. 4b,c). In addition, we analyzed the differential expression (Fig. 4e) of the genes commonly up- and downregulated in *hta9hta11* and *hta9hta11/FLAG-HTA9_N* or *hta9hta11/FLAG-HTA9_RR* (Supplementary Figure 4c). For this, up- or downregulated genes in *hta9hta11* were divided in four groups (200 to 300 genes in each group) according to their differential expression in *hta9hta11* relative to WT, and compared to the differential expression that they displayed in *hta9hta11/FLAG-HTA9_N* or *hta9hta11/FLAG-HTA9_RR*, as suggested by the Reviewer (see Fig. 4e). We found that the differential expression of the groups of downregulated genes was significantly reduced in both *hta9hta11/FLAG-HTA9_N* and *hta9hta11/FLAG-HTA9_RR* compared to *hta9hta11*, while the differential

expression of the groups of upregulated genes was only significantly reduced in *hta9hta11/FLAG-HTA9_N (*see results section, page 8-10 and the corresponding figures).

9. Line 262: "very high", put precise %.

Corrected

10. Line 277-282: This is not true, or at least, the figures provided (figure 4) do not show this. New figures need to be provided to re-examine the claims.

We have now provided new figures to sustain this claim (New Figure 4).

11. Line 291: no the results do not. The results about HTA9ub are only clear on the FLC and FT loci, but this is hardly enough to make sweeping claims. New analyses should be provided (as indicated above) to make proper claims about the function of HTA9ub in repression rather than activation.

We have now provided new figures to sustain this claim (New Figure 4).

12. Line 302: remove word "reduces"

Corrected

13. Line 310/311: the authors have not shown that the level of H2A.Z in genebody is a consequence of transcription. Part of the problem is that the claim that HTA9ub is on +1 does not hold, and so this may occur along the genebody, explaining the negative correlation. Secondly, the authors may have indicated (and with some refined analyses in the future one may say shown) that HTA9ub is important for H2A.Z mediated gene repression, but they did not show it is 100%. The construct with the mutated K has still half the effectivity of the non-mutated one (though again, this is based on an incorrect analysis) 19% compared to 36%, indicating that some repressive ability still exists. As it stands the authors can only say that modified HTA9 has a larger role in repression than unmodified HTA9.

We have now provided new analyses to sustain the repressive role of HTA9ub (New Figure 4). In addition, we found that despite both FLAG-HTA9_N and FLAG-HTA9_RR were similarly incorporated into chromatin (see new Fig. 6a), a much higher number of the upregulated genes in *hta9hta11* recovered or "partially recovered" their WT expression levels when FLAG-HTA9_N was incorporated (Fig. 4). Therefore, we propose that H2A.Zub has a repressive role and that H2A.Zub, more than the incorporation of H2A.Z by itself, is what is important to mediate the repression, at least of the upregulated genes in *hta9hta11*.

14. Figure 3b. It would be better to use a color gradient to indicate transcript amount. (like from light yellow for lowly expressed to dark red for highly expressed)

We did not follow reviewer suggestion in changing the color code since I personally have experienced difficulties in appreciating tonality changes in similar figures.

15. Figure 6c. Results of statistical tests missing.

We have changed this figure (see now new Fig. 6a). In the new one we have added statistical tests.

16. Sup fig 2A could use some annotation of the expected identity of the different bands

Corrected

17. Supplementary figure 4b. Results of statistical tests missing. And odd dots are present next to error bars that don't seem to have any purpose.

The bar plots were included to have some examples of the changes in expression levels observed in the different genotypes (obtained by RNA-seq and expressed in FPKM). The two dots represent the value of the different biological replicates. It is recommended that individual data from each experiment should be plotted if n < 5 alongside an error bar (Sullivan et al., 2016, Common Statistical Pitfalls in Basic Science Research). We have now stated what means the dots in the figure legend. In addition, we have now included statistical test of differentially expressed genes in the different genotypes in Figure 4e.

18. General: it would be good that the authors indicated which genes they included in their analysis. Whether they looked at all nuclear genes, or only protein-coding genes. And also how many genes were detected in the RNAseq experiments. Firstly it would be good to know in general on what kinds of genes the results are obtained from. But especially for the venn-diagrams it would be easier to determine how likely it is to obtain overlaps by chance (and whether there are over or under enrichments.

The number of genes scored as present in at least one of our RNA samples was 23,486, representing around 70% of Arabidopsis nuclear genes. For the analysis of H2A.Z enriched genes, and H3K27me3 and H2AK121ub marked genes in WT, all nuclear genes were considered. This information has been added in Methods section.

<u>Reviewer #3</u>

I must say I was a bit disappointed with the new version of the manuscript. The major change was removing the part of H2A.Z histone acetylation. The authors should know, however, that it is expected to add new data to the ms during revision, and not to remove them (though in this case this was suggested). Unfortunately, the authors added only one experiment (Fig. 6c), poor substitute for what was expected (see below). In my opinion, more work is required to warrant publication in Nature Communications.

I am not convinced at all with the way the authors tried to confirm +1 nucleosome location of H2A.Zub. I understand that @HTA9ub is not available, but it is possible to ChIP on BMI1 or at least H2AK121ub, not mentioning sequential ChIP on FLAG-HTA9 and then @Ub.

We think that the Reviewer comment indicating that the major change that we included in the new version was removing the part of H2A.Z histone acetylation (which was recommended by the three Reviewers) was a bit unfair. In the previous version we added a number of new figures to respond several Reviewers concerns (e.g. Fig. 1g;

Fig. 2c; Fig. 3c,d; Fig. 6c; Suppl Fig. 1b; Suppl Fig. 4a; Suppl Fig 6) and supplementary Data showing quantification of H2A.Z signal at different regions as an attempt to predict the localization of H2A.Zub. In addition, the manuscript text was substantially modified according to Reviewers indications.

In any case, we agree that the correlation between the amount of HTA9ub and the H2A.Z signal at +1 nucleosome does not represent direct evidence of the localization of H2A.Zub at +1 nucleosome region. The Reviewer proposed to ChIP on BMI1 or H2AK121ub or a sequential-ChIP experiment as alternative ways to determine HTA9ub localization in absence of an anti-H2A.Zub specific antibody. Unfortunately, none of these experiments will provide the desired information for the following reasons:

1) A ChIP on BMI1 will not address this question as BMI1 is involved in both H2A.Z and H2A monoubiquitination and we would not be able to differentiate if BMI1 is there to monoubiquitinate H2A or H2A.Z.

2) A ChIP on H2AK121ub will not work as well. We have already performed a ChIP-seq on canonical H2AK121ub (Zhou, Romero-Campero et al., 2017), however, this does not give information about the localization of H2A.Zub.

3) A sequential-ChIP, first using anti-HTA9 or -FLAG antibody and then anti-ubiquitin, will not provide a reliable outcome as we have to perform this experiment in a chromatin context in which nucleosomes are constituted by different histones that could also carry this modification. We have shown that canonical H2A can be monoubiquitinated by AtBMI1 to mediate gene repression (Bratzel et al., 2010; Yang et al., 2013; Zhou, Romero-Campero et al., 2017). Here, we showed that H2A.Z enriched genes overlapped with H2AK121ub marked genes (11,804 genes out of 22,523 H2A.Z enriched genes were H2AK121ub marked; Fig. 3d), indicating H2A.Z and H2AK121ub marks co-occupy a considerable number of genes. Since 80% of the H2AK121ub peaks overlap with the region immediately downstream the TSS of target genes (Zhou, Romero-Campero et al., 2017), it will be not possible to determine if the ubiquitin antibody is recognizing the mark incorporated into H2A.Z or into canonical H2A. Moreover, H2B can also be monoubiquitinated (Cao et al., 2008; Gu et al., 2009; Xu et al., 2009; Roudier et al., 2011). According to this, WB using anti-ubiquitin antibody on Arabidopsis histone extracts detected H2Bub, H2AK121ub and H2A.Zub (Fig. 2c). Sequeira-Mendes et al., 2014 defined several chromatin states in Arabidopsis based on the modifications and histone variants predominantly found in them. State 1 is characterized by high amounts of H3K4me3, H3 acetylation, H3K36me3, H2Bub and by nucleosome enriched in H3.3 and H2A.Z, which was typically associated with transcribed regions and TSSs. H2Bub and H2A.Z are also found in chromatin state 3, which is associated with transcriptionally elongating regions. Accordingly, H2Bub and H2A.Z may co-localize at different regions and thus, again, it will be not possible to determine if the ubiquitin antibody is recognizing the mark incorporated into H2A.Z or H2B.

In summary, we can IP chromatin containing HTA9 or FLAG-HTA9; however, in the second ChIP we will not be able to distinguish if the immunoprecipitated material resulted from the binding of the antibody to an ubiquitin residue present in HTA9 or in other histone. A reliable localization of H2A.Zub marks definitely requires a specific

antibody. Since this tool is not yet available, we decided to remove this part from the manuscript and just discussed briefly the possible localization of H2A.Zub in the discussion section (see page 15-16). We hope that the Reviewer will understand.

In the new Fig. 6c the authors compared the H2A.Z levels in two genes, FLC and HSP70, for which transcription H2A.Z has promoting and repressive effect, respectively. There are several problems with this experiment: First, there is no data that those genes are directly regulated by H2A.Z – one can imagine that reduced levels of H2A.Z in hta.z double mutant affect expression of some TFs, which in turn change FLC and HSP70 expression. Although FLC and HSP70 were extensively described in the context of H2A.Z, this was done 10 years ago or more, when our knowledge about H2A.Z role in transcriptional regulation was very limited, and direct effect was never proved for those genes. Therefore, the authors should include more than just one gene per each group, selecting genes from more recent works. Second, amplicons were not wisely chosen: the authors took amplicon +80 from Kumar & Wigge Cell 2010 paper instead of amplicon +119, which represents the center of +1 nucleosome, all other amplicons show even lower levels of H2A.Z, thus the signal may represent just a noise even when it is not observed in hta9 hta11 mutant shown in a supplementary file. In concordance with this, the second FLC amplicon selected by Gomez-Zambrano et al. lies in the middle of long intron and it is known to have no H2A.Z (see eg Deal et al. Plant Cell 2007), while the authors can still retrieve significant enrichment.

The negative control from Suppl. Fig. 6e should be presented along with the other line, otherwise it is misleading. Why the authors did not selected amplicons based on available browsers showing H2A.Z distribution in a genome-wide manner? I think that this experiment should be extended for other genes presented in Fig. 6b, providing that their also differ in transcriptional behavior in hta9 hta11 (or at least in arp6 mutant). Another option would be to add some genes extensively studied in the context of H2A.Z distribution, eg some genes presented in Sura et al. Plant Cell 2017 paper. This would provide an opportunity to extend their conclusions with the authors new data.

According to Reviewer suggestions, we have included more genes in the analysis, added browser views of H2A.Z localization at the selected genes with the exact location of amplicons, and included *hta9hta11* together with the other lines as a control. We have in addition normalized the data to *ACT7* to correct background signal (see new Fig. 6a). We would like to thank Reviewer for his/her suggestions as the figure has substantially improved. However, we would like to mention that we consider *HSP70* and *FLC* genes bona fide representative genes of H2A.Z mediated gene regulation. The result by Kumar and Wigge (2010) showing that in *arp6-10* there is a drastic reduction of H2A.Z deposition into *HSP70* and a subsequent upregulation is quite clear; In addition, Cortijo et al. (2017) verified *HSP70* as a H2A.Z regulated gene. Also, our expression results in complemented lines, the H2A.Z localization at *HSP70* in WT and the loss of HTA9 in *hta9hta11* mutant strongly support a role of H2A.Z in regulating *HSP70* expression. There are even more examples showing that *FLC* is directly regulated by H2A.Z deposition. PIE1, ARP6 and SWC6 play key roles in the deposition of H2A.Z on *FLC* chromatin. Mutations in the corresponding genes cause acceleration of flowering, mainly due to low *FLC* expression (Noh & Amasino, 2003; Martin-Trillo *et al*., 2006; Choi *et al*., 2007; Deal *et al*., 2007; Lazaro *et al*., 2008). More recently,

Sura et al. (2017) showed that a HTA11-tagged version is incorporated into *FLC* chromatin and is able to restore *FLC* levels in *hta9hta11*. Furthermore, a very recent report showed that YAF9A and B target *FLC* chromatin and regulate its expression by acetylation of H2A.Z (Crevillen et al., 2019). They found that HTA11-GFP is enriched at the same region of *FLC* that we investigated (*FLC-1*) and that the levels of HTA11-GFP were considerably reduces in *arp6* mutants. Our data also support a direct regulation of *FLC* by H2A.Z.

REVIEWERS' COMMENTS:

Reviewer #1 (Remarks to the Author):

The authors have satisfied all of my concerns about the manuscript.

Reviewer #2 (Remarks to the Author):

The authors have appropriately dealt with all major points of criticism. There are, however, a number minor issues to be addressed, which do not require further review.

Minor comments:
Line 94: cannot be ruled out

Figure 1e: visual artefacts

Figure 1e/f/g: I miss statistical comparisons between wt and transgenics (to test whether rescue has been achieved), and a comparison between RR/RK and mutant to tell whether they show any rescue at all)

Line 101-107: The text here should be improved to make it clearer. What you could say is that "all three lines partly rescue the early flowering phenotype of the hta9hta11 mutant, but the -N line shows a greater rescue than the RK and RR lines."

Line 105: test whether wt and hta9hta11 FLAG-HTA9N differ significantly.

Line 111: you did not test this.

Line 110: where->were

Line 128: 'the' Arabidopsis transcriptome

Line 130: 'considerable' is vague. Give a number or percentage.

Line 135: 'considerably' is vague. State how many fold the signal is reduced.

Line 154-156: sentence is not completely logical, I would rephrase: "the majority of the genes upregulated in hta9hta11 is also upregulated bmi1a/b, indicating that perhaps the H2A.Zub mark mediates this repression."

Line 162-165: I find that also here there is a logical problem, maybe wording can be improved. Another problem with this text and figure 3c is that the level of H2A.Z does not indicate the level of H2A.Zub. It can be that H2A.Z alone is enough to repress genes, or that a lower H2A.Z level is a consequence of expression. In fact, the last thing is what the authors even propose later on. I propose to leave out figure 3c.

Line 176: did the authors check the expression level of PRC1 subunits in the H2A.Z mutant? Maybe the increase in H2AK121ub can be explained by increased PRC1 activity?

figure 4d: I think it would be helpful to indicate in the figure (in the key) that yellow is low expression and blue is high expression. This is not immediately clear from the color (blue is usually low exression or downregulation), and from the value (negative values are usually linked to downregulation, not a low positive expression). I presume that some kind of normalization has been performed to put the average expression at 0, but this is not indicated anywhere.

Figure 4e: maybe I misunderstand the figure, but why are the boxplots belonging to the mutants (group1) not the same in the first and second panel?

Line 237: from

Line 247: do a test for significance and add result to 5a

Line 250-258: Because previous research (from the authors' lab) has shown that H3K27me3 dependent on PRC1 does not need to have H2Aub (the idea was that H2Aub may get removed after initial deposition), it means that one should also consider genes without H2A.Z. What the authors should then compare is genes upregulated in H2A.Z and PRC1 mutants, and genes marked with H3K27me3. Regardless of the comparison made, the authors ought to not only show the degree of overlap, but also indicate how much more or less that is than based on chance. If the authors are right, then they should show that the overlap (between genes with H3K27me3 and genes up in both mutants) is not higher than what is expected based on chance. Of course, the fact that the majority of upregulated genes are not marked with H3K27me3 is very informative by itself, but adding aforementioned data would be informative.

Line 263: were statistical tests performed? If so, indicate the results in figure 5c.

Line 270: 'few' is vague, give the percentage.

Figure 5d: perhaps add results of a hypergeometric test to determine whether the obtained overlap is lower than or equal to what is expected based on chance.

Supplemental figure 4a/b: results of statistical tests should be included

Line 311: text points to the wrong figures

Line 334: 'the' +1 nucleosome

Line 426/427: change to 'H2A.Z is involved in other processes'

Line 431: based on

Reviewer #3 (Remarks to the Author):

The manuscript has been significantly improved since the last round of the reviews. I understand the Authors arguments about the potential problems with interpretation of sequential ChIP experiments, which I found was suggested also by another reviewer and therefore accept the decision of removing the part about location of H2A.Zub in +1 nucleosome. I am quite satisfied with extending the number of genes in Fig. 6a, for which HTA9 deposition in different backgrounds was tested. I think that with this number of genes and improved way of their analysis and presentation, the conclusion about the importance of H2A.Z monoubiquitination for gene transcriptional repression is significantly better supported. I also liked many other changes which were suggested by the other reviewers, especially in the way the data were presented. I do not have more comments to the manuscript.

<u>Response to Reviewers</u>

Reviewer #1

The authors have satisfied all of my concerns about the manuscript.

Reviewer #2

The authors have appropriately dealt with all major points of criticism. There are, however, a number minor issues to be addressed, which do not require further review.

Minor comments:

Line 94: cannot be ruled out

Corrected

Figure 1e: visual artefacts

Sorry but I do not find the visual artefacts in figure 1e. In any case, we are now submitting the figures in other format and I hope this will resolve the problem.

Figure 1e/f/g: I miss statistical comparisons between wt and transgenics (to test whether rescue has been achieved), and a comparison between RR/RK and mutant to tell whether they show any rescue at all)

We have included the statistical comparisons. Although the analysis showed that the flowering time was significantly different in all the comparisons, the p-value of the comparison between WT and hta9hta11/FLAG-HTA9_N was 6-7 orders of magnitude higher than in the case of the other two lines. Similarly, the p-value of the comparison between hta9hta11 and hta9hta11/FLAG-HTA9_N was 7-8 orders of magnitude lower than in the case of the other lines. This confirm a partial rescue of the early flowering time in the three lines, showing -*N* line a greater rescue.

Line 101-107: The text here should be improved to make it clearer. What you could say is that "all three lines partly rescue the early flowering phenotype of the hta9hta11 mutant, but the -N line shows a greater rescue than the RK and RR lines."

Corrected

Line 105: test whether wt and hta9hta11 FLAG-HTA9N differ significantly.

As we indicated above, the difference between WT and hta9hta11/FLAG-HTA9_N was significant but less than in the case of the other two transgenic lines.

Line 111: you did not test this.

Tested and corrected

Line 110: where->were

Corrected

Line 128: 'the' Arabidopsis transcriptome

Corrected

Line 130: 'considerable' is vague. Give a number or percentage.

Corrected

Line 135: 'considerably' is vague. State how many fold the signal is reduced.

Corrected

Line 154-156: sentence is not completely logical, I would rephrase: "the majority of the genes upregulated in hta9hta11 is also upregulated bmi1a/b, indicating that perhaps the H2A.Zub mark mediates this repression."

Corrected

Line 162-165: I find that also here there is a logical problem, maybe wording can be improved. Another problem with this text and figure 3c is that the level of H2A.Z does not indicate the level of H2A.Zub. It can be that H2A.Z alone is enough to repress genes, or that a lower H2A.Z level is a consequence of expression. In fact, the last thing is what the authors even propose later on. I propose to leave out figure 3c.

We did not removed Fig. 3c but we have modified the text indicating that we found that in WT seedlings, the genes upregulated in *hta9hta11* and *atbmi1a/b* weak showed higher H2A.Z levels along the entire gene than the genes downregulated in *hta9hta11* (Fig. 3c), which was consistent with a transcriptionally repressed and activated state, respectively.

Line 176: did the authors check the expression level of PRC1 subunits in the H2A.Z mutant? Maybe the increase in H2AK121ub can be explained by increased PRC1 activity?

Yes, we checked but none of the AtBMI1 or AtRING1 genes passed out our cut-off criteria to be considered upregulated.

figure 4d: I think it would be helpful to indicate in the figure (in the key) that yellow is low expression and blue is high expression. This is not immediately clear from the color (blue is usually low exression or downregulation), and from the value (negative values are usually linked to downregulation, not a low positive expression). I presume that some kind of normalization has been performed to put the average expression at 0, but this is not indicated anywhere.

We have included this information in the figure legend. A standard normalization of the gene expression profiles was performed to obtain a mean expression of 0 and a standard deviation of 1 in order to make the expression profiles comparable.

Figure 4e: maybe I misunderstand the figure, but why are the boxplots belonging to the mutants (group1) not the same in the first and second panel?

This is because the genes that are commonly downregulated in *hta9hta11* and *hta9hta11/FLAG-HTA9_N* (first panel) are not exactly the same genes than the ones commonly downregulated in *hta9hta11* and *hta9hta11/FLAG-HTA9_RR* (second panel) and thus in hta9hta11 the distribution of the Log2(FC) is different in the two panels. This can be appreciate in group 1as this interval of Log2(FC) is bigger than in the other groups.

Line 237: from

Corrected

Line 247: do a test for significance and add result to 5a

Done

Line 250-258: Because previous research (from the authors' lab) has shown that H3K27me3 dependent on PRC1 does not need to have H2Aub (the idea was that H2Aub may get removed after initial deposition), it means that one should also consider genes without H2A.Z. What the authors should then compare is genes upregulated in H2A.Z and PRC1 mutants, and genes marked with H3K27me3. Regardless of the comparison made, the authors ought to not only show the degree of overlap, but also indicate how much more or less that is than based on chance. If the authors are right, then they should show that the overlap (between genes with H3K27me3 and genes up in both mutants) is not higher than what is expected based on chance. Of course, the fact that the majority of upregulated genes are not marked with H3K27me3 is very informative by itself, but adding aforementioned data would be informative.

We have previously shown that in Arabidopsis there is a subset of genes in which the deposition of H3K27me3 is dependent of H2Aub (around 5,000 genes) but also another subset of genes in which the deposition of H3K27me3 seems to be independent of H2Aub or AtBMI1A (around 1,800 genes), since H3K27me3 levels at these genes are not affected (Zhou, Romero-Campero et al., 2017). We do not really know whether H2Aub is initially incorporated at these genes and then removed or a different mechanism involving other PRC1 components works for the regulation of these genes. In any case, the AtBMI1s are involved in the modification of H2A.Z but not in the incorporation of H2A.Z variant, therefore, to focus the analysis on possible direct targets we have considered only genes with H2A.Z (which makes sense as if H2A.Z is not present it cannot directly affect gene expression). On the other hand, as this histone variant is widely distributed (22,523 genes), there are only 184 genes upregulated in *hta9hta11* and *atbmi1a/b/c* that are not marked with H2A.Z (Fig.3a). For the same reason, the percentage of genes with H3K27me3 and without H2A.Z is low (Suppl. Fig. 5a). Nevertheless, the comparison of genes upregulated in H2A.Z and PRC1 mutants and genes marked with H3K27me3 without considering H2A.Z, gives a similar result (31.4% of the upregulated genes were H3K27me3 marked and 68.5% were non H3K27me3 marked).

36% of overlapping genes in the comparison of Fig.5b is of course statistically significant; however, statistically significant p-values can be found if the sample size is large enough, therefore we have to evaluate whether this significant p-values are meaningful. The facts that the majority of upregulated genes are not marked with H3K27me3 (64%) and that the loss of PRC2 activity does not lead to the upregulation of the same subset of genes than the loss of H2A.Z support that H3K27me3 is dispensable for H2A.Z repressing effect, which is what we are claiming.
In any case, we have contemplated a possible role of H3K27me3 marks in reinforcing H2A.Zub mediated repression in the discussion section.

Line 263: were statistical tests performed? If so, indicate the results in figure 5c.
The results have been included

Line 270: 'few' is vague, give the percentage.
The percentage has been included

Figure 5d: perhaps add results of a hypergeometric test to determine whether the obtained overlap is lower than or equal to what is expected based on chance.
We found that only a 10% of the upregulated genes in each mutant overlapped in this comparison. This percentage, according to a hypergeometric test, is significant but again, we consider that the fact that 90% of the genes from each dataset do not overlap is more informative.

Supplemental figure 4a/b: results of statistical tests should be included
Done

Line 311: text points to the wrong figures
Corrected

Line 334: 'the' +1 nucleosome
Corrected

Line 426/427: change to 'H2A.Z is involved in other processes'
Corrected

Line 431: based on
Corrected

Reviewer #3
The manuscript has been significantly improved since the last round of the reviews. I understand the Authors arguments about the potential problems with interpretation of sequential ChIP experiments, which I found was suggested also by another reviewer and therefore accept the decision of removing the part about location of H2A.Zub in +1 nucleosome. I am quite satisfied with extending the number of genes in Fig. 6a, for which HTA9 deposition in different backgrounds was tested. I think that with this number of genes and improved way of their analysis and presentation, the conclusion about the importance of H2A.Z monoubiquitination for gene transcriptional repression is significantly better supported. I also liked many other changes which were suggested by the other reviewers, especially in the way the data were presented. I do not have more comments to the manuscript.
